# An Interspecific Fungal Hybrid Reveals Cross-Kingdom Rules for Allopolyploid Gene Expression Patterns

**DOI:** 10.1371/journal.pgen.1004180

**Published:** 2014-03-06

**Authors:** Murray P. Cox, Ting Dong, GengGeng Shen, Yogesh Dalvi, D. Barry Scott, Austen R. D. Ganley

**Affiliations:** 1 Institute of Fundamental Sciences, Massey University, Palmerston North, New Zealand; 2 Institute of Natural and Mathematical Sciences, Massey University, Auckland, New Zealand; University of California-Riverside, United States of America

## Abstract

Polyploidy, a state in which the chromosome complement has undergone an increase, is a major force in evolution. Understanding the consequences of polyploidy has received much attention, and allopolyploids, which result from the union of two different parental genomes, are of particular interest because they must overcome a suite of biological responses to this merger, known as “genome shock.” A key question is what happens to gene expression of the two gene copies following allopolyploidization, but until recently the tools to answer this question on a genome-wide basis were lacking. Here we utilize high throughput transcriptome sequencing to produce the first genome-wide picture of gene expression response to allopolyploidy in fungi. A novel pipeline for assigning sequence reads to the gene copies was used to quantify their expression in a fungal allopolyploid. We find that the transcriptional response to allopolyploidy is predominantly conservative: both copies of most genes are retained; over half the genes inherit parental gene expression patterns; and parental differential expression is often lost in the allopolyploid. Strikingly, the patterns of gene expression change are highly concordant with the genome-wide expression results of a cotton allopolyploid. The very different nature of these two allopolyploids implies a conserved, eukaryote-wide transcriptional response to genome merger. We provide evidence that the transcriptional responses we observe are mostly driven by intrinsic differences between the regulatory systems in the parent species, and from this propose a mechanistic model in which the cross-kingdom conservation in transcriptional response reflects conservation of the mutational processes underlying eukaryotic gene regulatory evolution. This work provides a platform to develop a universal understanding of gene expression response to allopolyploidy and suggests that allopolyploids are an exceptional system to investigate gene regulatory changes that have evolved in the parental species prior to allopolyploidization.

## Author Summary

Organisms are complex biological systems that must continue to function even as their genomes evolve. While evolution is usually gradual, the formation of new species by the hybridization of different parents—allopolyploidization—occurs nearly instantaneously. A key question is what happens to expression of the two parental gene copies following genome merger. To determine this, we focused on a fungal allopolyploid from a group that dominates many of the world's pastoral economies. To investigate the fate of gene expression in this system, we developed a novel pipeline to assign high throughput RNA sequence reads to the two parental gene copies, thus allowing quantification of expression. We found transcriptional responses to be predominantly conservative: most gene copies either inherit parental expression patterns, or if differentially expressed in the parents, that difference is lost in the hybrid. Moreover, we identified an extraordinary level of concordance in the fate of genome-wide allopolyploid gene expression with that seen in cotton. The very different nature of these two allopolyploids suggests that there is a set of universal rules for the transcriptional response to genome merger. We propose a mechanistic model whereby this conserved response reflects similarities in mutational processes that underlie gene regulatory evolution.

## Introduction

Polyploidization refers to events that result in a sudden increase in the number of chromosome sets carried by an organism. Polyploidy is a major force in evolution, and has led to the emergence of new lineages in many major eukaryotic groups [1]–[8]. Unlike the incremental series of small changes that characterize the usual evolutionary process, polyploidization has the potential to form new species nearly instantaneously [9]. There are two classes of polyploidization: autopolyploidy is the duplication of a genome; while allopolyploidy is caused by interspecific hybridization between different species or genera resulting in the union of two or more dissimilar genomes. Such allopolyploids are often ecologically competitive, in many cases showing improved adaptability relative to parental species [10]. This is thought to arise from masking of deleterious mutations, fixed heterosis (‘hybrid vigor’), and/or greater evolutionary plasticity resulting from the duplicated gene copies [11]–[13].

Genome shock describes changes in genome organization and behavior that occur in response to the sudden appearance of multiple genome copies [14]. Several manifestations of genome shock as a consequence of polyploidization are known, including gene loss, chromosome mis-pairing, transposon activation, altered methylation, and rearrangements between the genomes [1], [11], [15]–[21]. Gene loss has been particularly well studied in the hemiascomycetous yeasts, where substantial loss of gene duplicates has occurred following a whole genome duplication [22]–[24]. Gene loss has also been observed in a number of plant polyploids [2], [25]–[27] suggesting that it is a general feature of polyploidy. Nevertheless, some plant polyploids, such as cotton, retain remarkably stable parental genome complements [28], [29]. Moreover, different classes of genes are more prone to duplicate loss or retention following changes in ploidy [30], although the overall trend is great malleability in genomic responses to polyploidization.

**Figure 1:**
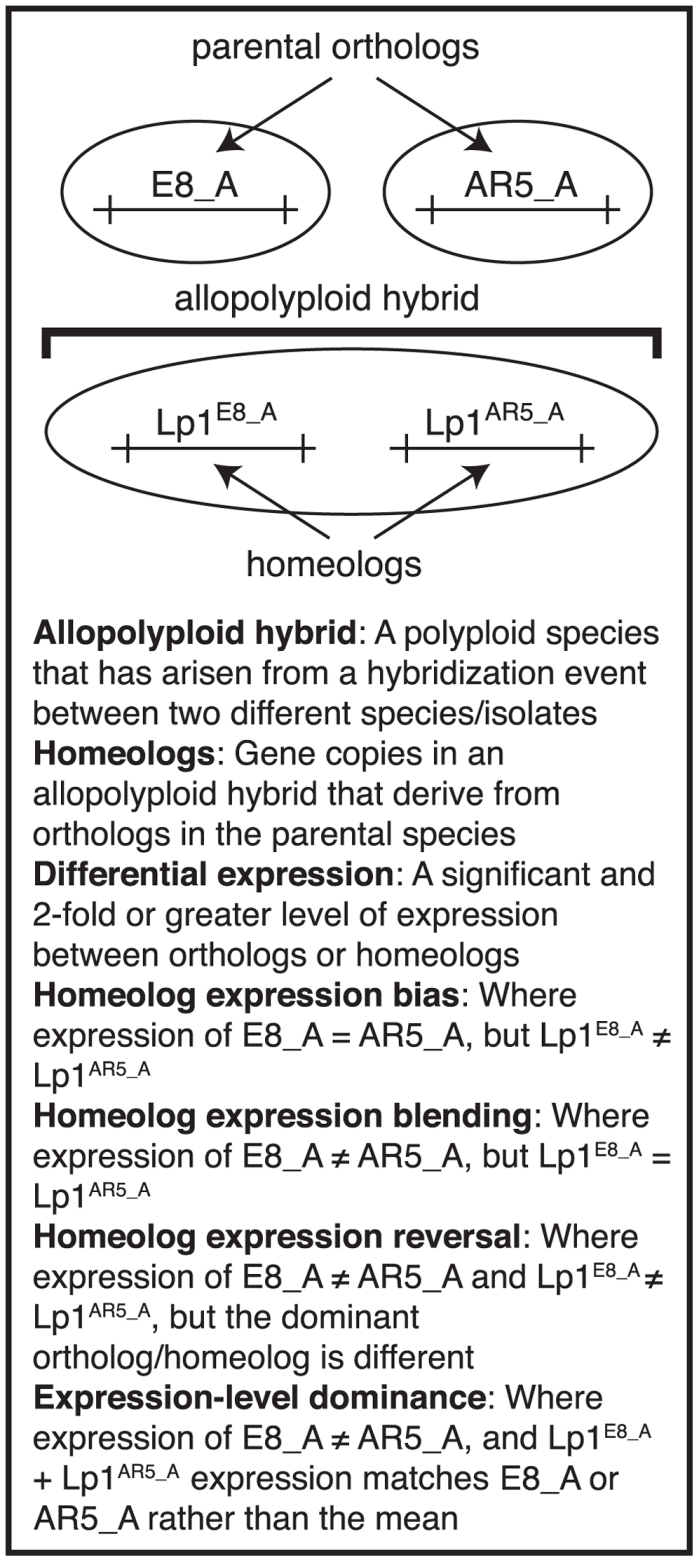
Glossary of terms. Diagram outlining the meanings of terms relating to allopolyploid transcription that are used in this study, and which derive from Yoo *et al.* [46]. Illustrated is an example of a gene, “A”, that is present as orthologs in the parents E8 and AR5, and as homeologs in the allopolyploid Lp1. The three homeolog expression terms are possible outcomes for relative gene expression in the allopolyploid. Homeolog expression bias: differential expression is not found between the two orthologs, but has arisen in the allopolyploid [43]. Homeolog expression blending: expression differences between the two orthologs have been lost in the allopolyploid. Homeolog expression reversal: the highly expressed ortholog has lower expression than the other homeolog in the allopolyploid.

Another feature of allopolyploids that has generated great interest is transcriptome shock, the sudden change in gene expression following the mixing of two dissimilar genomes, each with their own set of transcription factors and their own chromatin profiles [6], [31]. To date, most studies that have examined the response of gene expression to allopolyploidization have focused on plant systems, ranging from evolutionarily old allopolyploidy events through to synthetic plant allopolyploids [32]–[40]. Two transcriptional phenomena are emerging from these studies [41]–[43]. The first is called “homeolog expression bias” (here we use the term homeolog for the different parental copies of a gene within an allopolyploid; see Figure 1). This refers to cases where the homeologs in the allopolyploid show an expression pattern different to that observed in the parents. The second phenomenon, termed “expression-level dominance”, is where a gene that shows a difference in expression between the two parents has a combined level of expression from the two homeologs in the allopolyploid that resembles one of the parental expression levels, rather than being an average of the two. However, because the single gene methods and probe-based assays (such as microarrays) traditionally used to study allopolyploid expression lack sufficient resolution to reveal the full suite of genome-wide gene expression patterns at an individual homeolog level [44], the generality of these phenomena and how they are manifested has not been clear. High throughput mRNA sequencing technologies can overcome these limitations by resolving the expression levels of each homeolog in allopolyploids [45]. Recent studies have started to utilize these sequencing technologies to address allopolyploidization, and these are beginning to reveal the complex and multi-faceted transcriptional changes that occur during and after this event [39], [45], [46].

While much research has focused on plants, polyploidization is also an important feature of the evolutionary history of other lineages, including the fungi [47]–[49]. A model system for studying fungal allopolyploidy is the epichloë endophytes, an ecologically and economically important group of fungi from the family Clavicipitaceae [50] that includes the genera *Epichloë* and *Neotyphodium*. As systemic, obligate symbionts of cool-season grasses, the epichloë endophytes underpin the productivity of most global pastoral economies [51]. Most epichloë endophytes produce a range of biologically active secondary metabolites that can be economically beneficial by protecting against insect damage, but can also be economically detrimental by preventing mammalian herbivory, resulting in livestock productivity losses through toxic conditions called ryegrass staggers and fescue toxicosis. Interestingly, many species are natural polyploids [5], [52], which are usually referred to as “hybrids” in the fungal literature.

Epichloë polyploids are often formed from very different parents, with nucleotide divergence between parent species reaching as high as 8% [49], a level of divergence comparable to that between humans and macaques [53]. One well-characterized epichloë polyploid, designated as Lp1, is an economically important asexual interspecific hybrid (hereafter referred to as an allopolyploid to conform with the extensive plant literature) between a haploid sexual species, *E. typhina*, and a haploid asexual species, *N. lolii* [50], [54]–[56]. Although the mechanism of allopolyploidization is not known, it is thought to be similar to normal fungal mating, where cells first fuse to create a dikaryon (a cell containing two nuclei), followed by nuclear fusion [49]. Lp1 cells have been shown microscopically to contain only a single nucleus [50], proving that it is a true allopolyploid and not simply a fungal dikaryon, and because Lp1 is asexual, this nuclear arrangement is a permanent state. The allopolyploid nature of Lp1 is reinforced by genetic data: most genes studied to date in Lp1 have both parental copies maintained [50], [54], with the notable exception of the ribosomal DNA repeats (rDNA) and the mitochondrial DNA, each of which derive from just one parent [54], [55], [57]. The existence of species closely related to the original parents [50], coupled with it being a naturally occurring allopolyploid, make Lp1 an ideal system to explore the consequences of allopolyploidy on transcription within the fungi.

Here, we describe an RNA-seq based analysis of gene expression in the Lp1 allopolyploid. We develop a new computational pipeline to determine gene expression levels of both homeologs using next-generation mRNA sequencing in Lp1 without the benefit of a close reference genome sequence, and compare these to the parental species' transcriptomes. We document the four possible relative gene expression level outcomes for orthologs that become united in an allopolyploid, as well as a total expression level outcome, expression-level dominance (Figure 1). Our results show remarkable concordance of allopolyploid gene expression outcomes with those seen in plants, suggesting there are common transcriptional responses to allopolyploidy that reflect the underlying systems of gene regulation. We also show that almost all genes are retained in duplicate in Lp1, implying that little gene loss has occurred since the polyploidization event, which we calculate occurred no more than 300,000 years ago.

## Results

### Assigning mRNA sequences in the allopolyploid to parental homeologs

**Figure 2:**
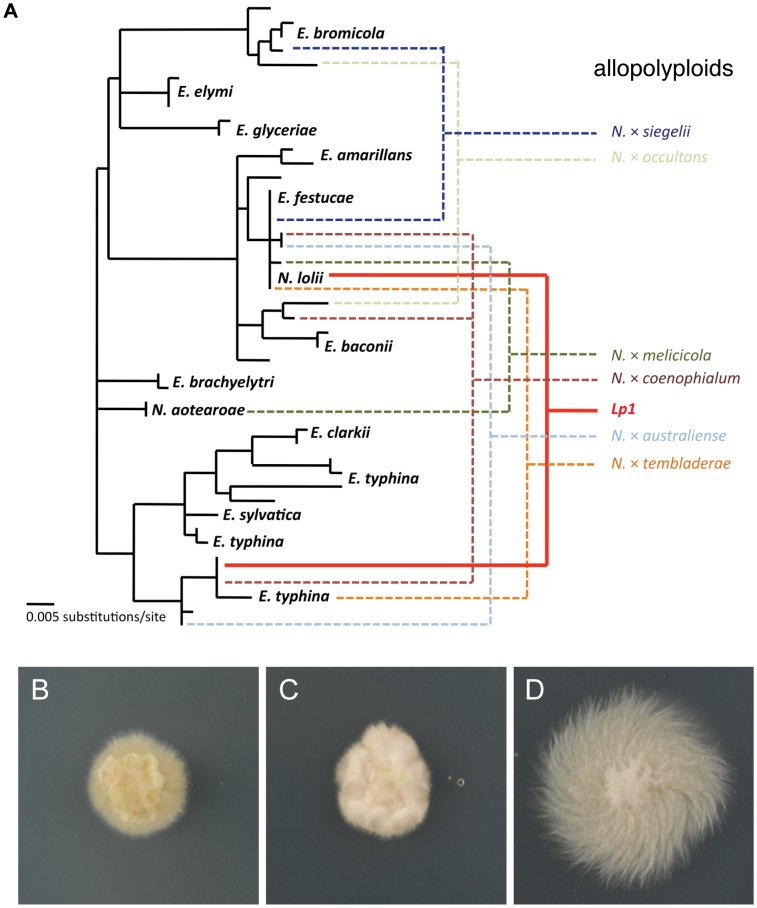
Phylogeny and culture morphology of *Epichloë* and *Neotyphodium* species. (A) Phylogeny of *Epichloë* and *Neotyphodium* fungi. Haploid species are indicated in black, while some of the many natural allopolyploid species in these genera are shown to the right. Colored lines indicate the parents of these allopolyploids, including the ancestors of the allopolyploid investigated in this study, Lp1 (solid red line). Phylogeny modified from published sources [49], [51], [82]. Culture morphology of (B) *N. lolii* AR5, (C) allopolyploid *N. lolii*×*E. typhina* Lp1 and (D) *E. typhina* E8. All culture panels 30 mm square.

To investigate the fate of gene expression following allopolyploidization in the well-characterized *Neotyphodium lolii*×*Epichloë typhina* allopolyploid endophyte, Lp1, we performed Illumina mRNA sequencing on Lp1 and its putative parental species (Figure 2). The closest extant *E. typhina* strain is believed to be E8 [50]. Previous studies employed strain Lp5 as the closest *N. lolii* parent [e.g., 57]. However, in this study we used *N. lolii* strain AR5 as the parental isolate because (a) it was isolated from the same germplasm as Lp1 (B. Tapper, AgResearch New Zealand, pers. comm.), and (b) AR5 and the *N. lolii* component of the Lp1 genome are identical over a 430 bp region of the *PYR4* gene (results not shown).

Replicate cultures of Lp1, AR5 and E8 were grown on rich medium and then transferred to a defined nutrient-limited medium to stimulate transcription of a wide range of genes [58]. After 24 h of growth, mRNA was extracted from culture mycelium and sequenced using Illumina HiSeq sequencing. The numbers of reads obtained are shown in Table 1. As a quick validation of data quality, reads were mapped to the E2368 reference genome. Mapping rates varied between 66–80%, with most unmapped reads subsequently identified as rRNA derived.

**Table 1:** Read counts from Illumina sequencing of Lp1 and its parents.

Strain	Read Type	Biological Replicate A	Biological Replicate B
AR5	Single-end	35,333,897	36,358,640
E8	Single-end	33,627,200	27,209,743
Lp1	Paired-end	93,408,176	62,327,160

To allocate Lp1 reads to the AR5- and E8-like homeologs, we developed a pipeline (Figure S1) that utilizes an existing catalogue of well annotated gene models developed from the genome sequence of a related epichloë species, *E. festucae* strain E2368 [59]. We created two separate reference gene sets, one each for the AR5- and E8-like homeologs. To achieve this, we first identified AR5- and E8-specific SNPs by mapping reads from the two parental species to the E2368 gene models at low stringency. Relative to E2368, we identified 44,665 and 336,802 SNPs for the AR5 and E8 parental species, respectively. This order-of-magnitude difference reflects the close evolutionary relationship between AR5 and E2368, both of which have an *E. festucae* ancestry, compared to the more divergent *E. typhina* strain E8. The median distance between SNPs in AR5 was 244 bp (95% confidence interval: 3–1,725 bp), while the median distance between SNPs in E8 was 22 bp (95% CI: 1–141 bp). Because Lp1 carries additional SNPs (*i.e.*, polymorphisms that have arisen since the allopolyploidization event), we also mapped the Lp1 reads at low stringency to the E2368 gene models. 374,931 SNPs were identified, most of which (94%) were shared with one or other of the parent species. Of the 22,543 new SNPs identified, 3,241 (14%) could be assigned to either the AR5 homeolog (1,745) or the E8 homeolog (1,496) on the basis of linkage disequilibrium with known AR5 and E8 parental SNPs. All SNPs (*i.e.*, those in both Lp1 and its parents) were divided into diagnostic classes that are informative for determining homeolog ancestry (Table 2) and these were used to create the two reference gene sets. After culling genes with insufficient sequencing coverage (Protocol S1), the reference gene sets contained 6,698 (55%) of the 12,199 predicted gene models from the E2368 strain reference.

**Table 2:** SNP counts by diagnostic class.

SNP Class	SNP Count
Ancestral	10,730
AR5-unique	2,183
E8-unique	55,024
AR5-shared	13,065
E8-shared	260,229
Lp1-unique*	22,543
Lp1-AR5*	1,745
Lp1-E8*	1,496
Lp1-unclassified*	19,302

* Lp1-unique incorporates classes Lp1-AR5, Lp1-E8 and Lp1-unclassified.

We used this SNP dataset to genetically date the allopolyploidization event, with the results suggesting this occurred within the last 300,000 years (see Text S1). This first estimate of the age of Lp1 suggests that it is comparatively young, at least compared to the better-known autopolyploid event in hemiascomycetous yeast that occurred millions of years ago [22].

### Persistence of both parental gene copies in the allopolyploid

The two biological replicate datasets from Lp1 were mapped separately to the AR5- and E8-like reference gene sets with high stringency (*i.e.* zero mismatch mappings) to determine from which homeolog each read originates. This resulted in 12,283,690 reads mapping uniquely to the AR5-like reference, and 11,769,529 reads mapping uniquely to the E8-like reference.

Previous investigations on just ten genes suggested that, apart from the special cases of rDNA and mtDNA (see Text S1), Lp1 contains both parent genomes with little evidence of gene loss [50], [54]. To explore gene loss on a genome-wide scale, we determined the number of genes with reads mapped to the respective AR5- and E8-like homeologs. Of the 6,698 genes for which we are able to distinguish homeologs, at least one read mapped to 6,654 genes (99.3%), thus under our study conditions only 44 genes had no detectable expression of either homeolog in Lp1. In addition, 35 genes showed expression solely from the AR5 homeolog, while 8 genes showed expression solely from the E8 homeolog. Therefore only 87 genes are candidates for gene loss events (see later), suggesting that Lp1 has not experienced widespread gene loss following allopolyploidization.

### Characterization of genes showing extreme differential expression

Genes that have the largest expression differences in Lp1 may represent biologically important functions that can shed light on the adaptive response of gene expression to allopolyploidization. We defined extreme differentially expressed (EDE) genes as those with very large expression differences in the allopolyploid – either a 50-fold or greater difference in expression level, or no expression from one of the parental homeologs. Excluding genes with fewer than 5 reads in the Lp1 dataset, 58 genes (0.9%) fit these criteria. To test for common functions in EDE genes, we performed a gene ontology (GO) slim analysis to determine whether certain classes of genes identified in the analyses above were enriched for particular functional categories. No strong patterns were detected (results not shown), although this outcome is tempered by the small number of genes analyzed.

**Figure 3:**
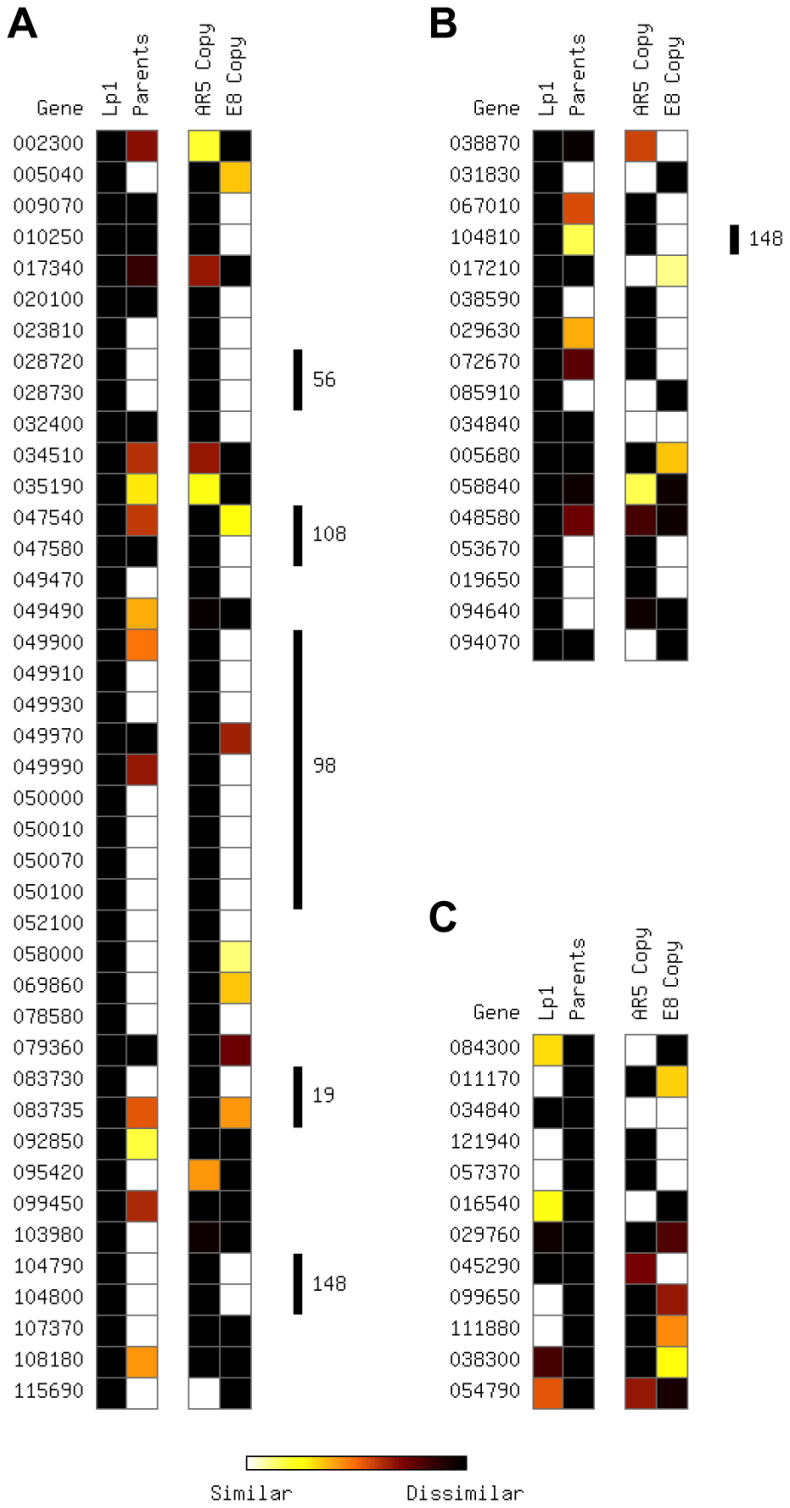
Parental and allopolyploid expression patterns from extreme differentially expressed genes. Plots show the relative expression level between the two homeologs in Lp1 (“Lp1”), between AR5 and E8 orthologs (“Parents”), between the AR5 ortholog and the AR5-like Lp1 homeolog (“AR5 copy”), and between the E8 ortholog and the E8-like Lp1 homeolog (“E8 copy”). (A) Genes with zero expression for one Lp1 homeolog, but non-zero expression for the other; (B) genes with >50-fold difference in expression between the Lp1 homeologs; (C) genes with >50-fold difference in expression between AR5 and E8 orthologs. Heat spectrum (bottom) varies between similar expression (white) and different expression (black). Vertical bars to the right indicate where genes are physically clustered; these are labeled by supercontig number. Genes on supercontig 148, present in (A) and (B), are physically contiguous in the genome. Genes 049470 and 049490, despite their numbering, are not physically adjacent.

To determine whether EDE gene expression patterns result from allopolyploidy or instead derive from regulation differences that exist between the parents, we constructed a heat map showing the ratio of gene expression levels between homeologs in Lp1 and the corresponding orthologs in the AR5 and E8 parents (Figure 3A and 3B). Expression ratios were determined in two ways. First, we calculated expression ratios between the two homeologs in Lp1, and between the corresponding AR5 and E8 parental orthologs (the two left-hand columns) to determine whether the biased expression observed in the allopolyploid is also present in the parents. Second, we calculated expression ratios between the AR5 parental ortholog and the AR5 homeolog, and between the E8 parental ortholog and the E8 homeolog (the two right-hand columns), to determine which homeolog is responsible for any expression change. Interestingly, about half the EDE genes where only one homeolog is expressed in Lp1 (21 of 41 using a 2-fold difference in expression as the cutoff) are due to changes in the allopolyploid (Figure 3A). A similar picture is seen for EDE genes with >50-fold expression difference between homeologs in Lp1 (Figure 3B). In both cases most expression changes have occurred in the AR5 homeolog.

For completeness, we also looked at the reciprocal situation: determining the fate of genes with high expression differences between the E8 and AR5 parents (Figure 3C). Only 12 orthologs showed 50-fold or greater difference in expression between the AR5 and E8 parents (our mapping approach automatically excludes any genes with expression in only one parent). Again, for about half of these genes (5/12), the differential expression is lost in Lp1.

### Deletions, rather than regulatory changes, explain major differences in homeolog expression

Loss of gene expression from one homeolog in the allopolyploid may result from chromatin changes or gene deletion, and may affect a broader genomic region than just a single gene. We therefore looked to see whether the EDE genes are physically linked in the reference genome [59]. Strikingly, 18 of 59 EDE genes (30.5%) are found in five physically contiguous clusters of two or more genes (Figure 3A,B). This pattern is particularly common in genes where only one homeolog is expressed in Lp1, with almost half of these genes occurring in clusters. To distinguish between these regions becoming heterochromatinized, thus silencing blocks of genes, or being a result of genomic deletions, PCR-RFLP of genomic DNA was employed.

**Figure 4:**
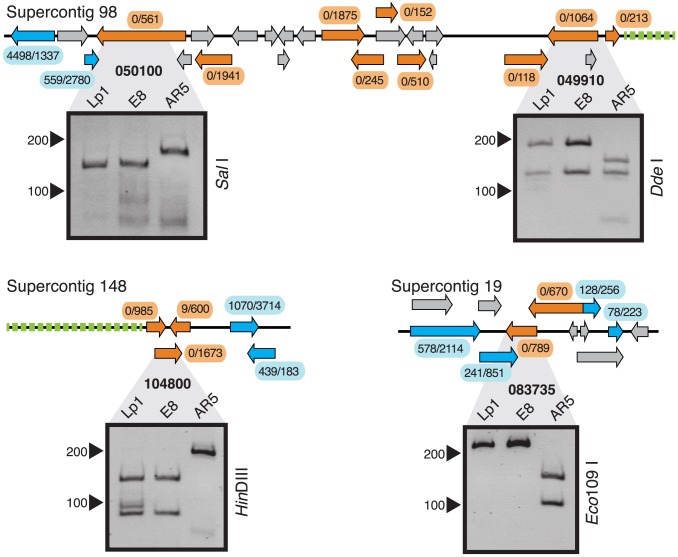
Gene loss in clustered extreme differentially expressed genes in Lp1. Three clusters of genes with extreme differential expression are shown; the supercontig from which they derive is indicated above. Orange arrows represent genes with extreme differential expression; blue, genes not showing extreme differential expression; and grey, genes culled by our mapping criteria (no expression information). Raw read numbers from AR5 and E8 homeologs, respectively, are shown in ellipses near each gene. Green dotted lines represent AT-rich regions. Below the genomic maps are PCR-RFLP results (reverse images of ethidium bromide-stained polyacrylamide gels) from Lp1, E8, and AR5 genomic DNA. The gene is indicated in bold above, the restriction enzyme to the right, and size marker positions to the left.

We chose three examples of clustered EDE genes to investigate (Figure 4). In one region, a block of nine genes on Supercontig 98 all show exclusively E8 homeolog expression in Lp1. PCR-RFLP analysis of the first and penultimate genes in this cluster shows that the AR5 homeolog has been deleted in both cases, and the most parsimonious interpretation is that a single, large deletion encompassing this entire block of genes has occurred in the AR5-derived genome. In the second example, a cluster of three genes is located on Supercontig 148, two of which show E8 homeolog-specific expression and one shows highly biased E8 homeolog expression. PCR-RFLP analysis indicates that the AR5 homeolog of the middle gene in this block has been deleted. We propose that this three-gene cluster has been deleted from the AR5-derived genome, with some reads emanating from a fragment of the weakly-expressed AR5 homeolog that remains. Interestingly, both these deletion events are adjacent to AT-rich regions at the end of the contig, which are usually indicative of transposon-rich repeat regions in epichloë species [59]. The third example is a cluster of two genes on Supercontig 19 that show E8 homeolog-specific expression. PCR-RFLP analysis also indicates deletion of the AR5 homeolog. Additionally, PCR-RFLP on non-clustered EDE genes shows that some, but not all, are deleted (Figure S2). These results illustrate that gene loss accounts for some of the most extreme homeolog expression biases observed in Lp1, and suggests that at least 20 genes have been deleted from Lp1 since the allopolyploidization event. The true number is likely to be larger, as biased homeolog expression from partial gene deletions is difficult to diagnose, and because our mapping procedure excluded about half the genes in the genome. This pattern of clustered gene loss resembles the chromosomal deletions observed in allopolyploid species of the plant genus *Tragopogon* [26], [60].

### Allopolyploidy leads to expression conformity of homeologs

Given the low level of gene loss, we next investigated the fate of gene expression following the allopolyploidization event on a genome-wide basis. The enormous number of reads in our study gave us great statistical power, meaning that for highly expressed genes a small proportional difference in read counts is statistically significant. Therefore we also employed a biological criterion of a 2-fold difference in expression between the AR5- and E8 homeologs (after normalization for different numbers of reads per sample) as a conservative cut-off for differential expression. Surprisingly, the majority of genes (4,515; 67.4%) did not differ from the null expectation of equal expression of the two homeologs in Lp1.

**Figure 5:**
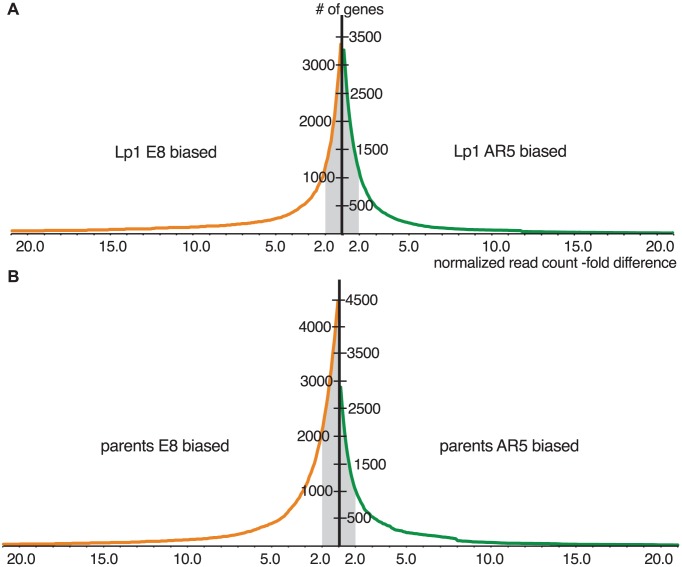
Cumulative distributions of gene expression ratios for Lp1 and parents. Plotted are the cumulative distributions of genes by normalized gene expression ratio between homeologs in Lp1 (A), and between orthologs from the parents (B). Those with higher expression from the E8-derived copy are shown in orange (left); higher AR5-derived expression in green (right). Grey shading indicates the pool of genes with less than 2-fold expression difference between copies (not differentially expressed). The bias towards more orthologs in E8 having greater relative expression can be seen in (B).

The above analysis ignores the relative expression level of the parental orthologs. To compare gene expression between the allopolyploid and the parental species, we calculated the relative expression levels of orthologs from the parental transcriptome data (cultured at the same time under identical conditions). We plotted the cumulative distribution of gene expression ratios between the AR5 and E8 homeologs in Lp1, and between the parental orthologs, to reveal how expression differences between homeologs/orthologs are distributed (Figure 5). Figure 5A shows that the majority of homeologs in Lp1 are not differentially expressed. Furthermore, there is no trend towards higher expression of one homeolog over the other: there are similar numbers of genes where the E8 homeolog is dominant and where the AR5 homeolog is dominant. In other words, Lp1 exhibits balanced homeolog expression bias [43]. In contrast, comparison of the parental species shows that more orthologs have higher relative expression in E8 than AR5 (Figure 5B), particularly genes that show relatively minor expression differences (2–10 fold). This pattern is offset by a small proportion of genes that have much higher relative expression in AR5, thus balancing total normalized read counts between E8 and AR5. Together, these results suggest that the predominant transcriptional response to allopolyploidy in Lp1 has been an overall reduction in differential homeolog expression relative to the parent species.

**Figure 6:**
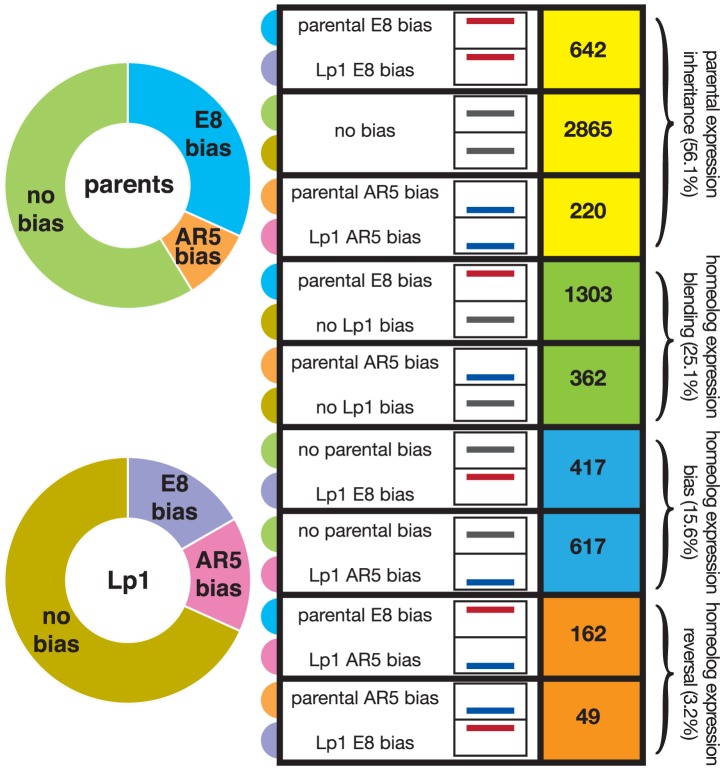
Fate of gene expression in the allopolyploid compared to the parents. Relative expression of the AR5-derived and E8-derived gene copies was calculated for Lp1 and its parents; ‘biased’ is 2-fold or greater expression difference. The right indicates numbers of genes in each of the nine possible combinations of biased/non-biased expression [46]. Box-graphs represent relative expression ratios, with the position of the line within the box indicating the E8:AR5 expression ratio. Specifically, red lines high in the box indicate E8 homeolog/ortholog expression bias, black lines mid-way in the box indicate equal expression, and blue lines low in the box indicate AR5 bias. These are grouped into four expression response categories: maintenance of the parental expression pattern in Lp1; homeolog expression blending; homeolog expression bias; and homeolog expression reversal, indicated by the colored boxes on the right. On the left are proportions of genes showing bias towards AR5 or E8 expression or no bias for the parents (top) and Lp1 (bottom). The colored half-circle tabs link the nine expression combinations to the plots on the left.

To investigate the fate of gene expression in Lp1 further, we used the framework developed in cotton [41], [46], where genes are divided into different classes representing their transcriptional response following allopolyploidy. For this analysis, the key is a comparison of the expression ratio between the two orthologs in the parent species with that of the two homeologs in the allopolyploid, and thus looks at relative transcriptional responses. The E8-like gene can either show higher expression, lower expression, or similar expression to the AR5-like gene, both between orthologs in the parents, and between homeologs in Lp1. The nine possible combinations of these states that derive from Table 3 of Yoo *et al.* [46] are shown in Figure 6. We binned each gene into one of these nine categories, again using a 2-fold difference in expression ratio as the cut-off.

**Table 3:** Similarities in the transcriptional response to allopolyploidy in Lp1 and natural cotton allopolyploids.

Expression response^a^	Lp1	Cotton^b^
Parental expression inheritance	56.1%	62.7–64.0%
Homeolog expression blending	25.1%	18.3–19.9%
Homeolog expression bias	15.6%	13.4–16.2%
Homeolog expression reversal	3.2%	2.6–2.9%
Expression-level dominance^c^	74.6%	73.9–75.1%
Non-dominant homeolog changing expression in expression-level dominance^d^	74.1%	76.3–78.3%

a Categories as presented in Figures 1, 6, and 7 [46].

b Ranges reported are those of the two natural allopolyploid cotton varieties analyzed in [46].

c Expressed as a percentage of all genes showing differential expression in the parents, including those with transgressive expression in [46].

d Expressed as a percentage of all genes showing expression-level dominance.

To analyze these results we used the scheme of Yoo *et al.*, who grouped the nine possible combinations of states (shown in Figure 6) into three broad outcomes for relative gene expression responses in the allopolyploid [46]. We put genes that show a reversal in the dominant homeolog into a separate group, thus giving four outcomes for gene expression that are defined in Figure 1. The first outcome (that includes the first three categories from Figure 6) is no change in expression patterns of AR5- and E8-like copies between the parents and the allopolyploid (for example, if a gene has greater expression in one parent, this pattern is maintained in the allopolyploid), something we call “parental expression inheritance”. A majority (56.1%) of genes display this behavior, with most being genes that show no expression difference between homeologs or orthologs. The second outcome contains genes displaying differential expression in the parents that has been lost in the allopolyploid. We call this ‘homeolog expression blending’, and about one-quarter (25.1%) of all genes show this pattern. The third outcome is the opposite pattern, homeolog expression bias, where differential expression has arisen in the allopolyploid [43]. The number of genes exhibiting homeolog expression bias is fewer (15.6%) than those showing homeolog expression blending. These results again illustrate the tendency towards reduced expression bias in the allopolyploid, and corroborate the picture obtained from the global analysis above. The final outcome is where an expression bias in the parents has been reversed in the allopolyploid (“homeolog expression reversal”). Unsurprisingly this outcome is relatively rare (3.2% of genes).

We next wondered whether the genes with altered expression patterns in the allopolyploid are responding to natural selection. We performed a GO-slim analysis on genes in the four outcome groups described above, as well as for all genes showing allopolyploid differential expression (Figure S3 and Text S2). No strong patterns of gene classes preferentially changing their transcription levels were identified, suggesting that if selective forces have shaped the transcriptional response in Lp1, it has only been for a small minority of genes.

### Lp1 shows expression-level dominance driven by change of the non-dominant homeolog

Recent studies have found evidence for expression-level dominance in allopolyploids, where genes that show differential expression between the parents have a total combined homeolog expression level that is similar to one or other of the parental levels (the ‘dominant’ ortholog) in the allopolyploid, rather than simply approaching the average of the orthologs [37], [41], [42], [46]. To investigate whether this phenomenon also occurs in Lp1, we calculated total expression for each gene in Lp1 that had 2-fold or greater expression difference in the parents, and classified these genes into three bins where allopolyploid expression more closely resembles: the highly-expressed ortholog; the lowly-expressed ortholog; or the mean of the orthologs (Figure 7). Almost three quarters of genes that show differential expression in the parents had an expression level similar to one ortholog (the dominant ortholog). Interestingly, a similar number of genes matched the low expression ortholog as matched the high expression ortholog. Therefore, in keeping with other studies, we find that expression-level dominance is a major factor in the transcriptional response to allopolyploidy in Lp1.

**Figure 7:**
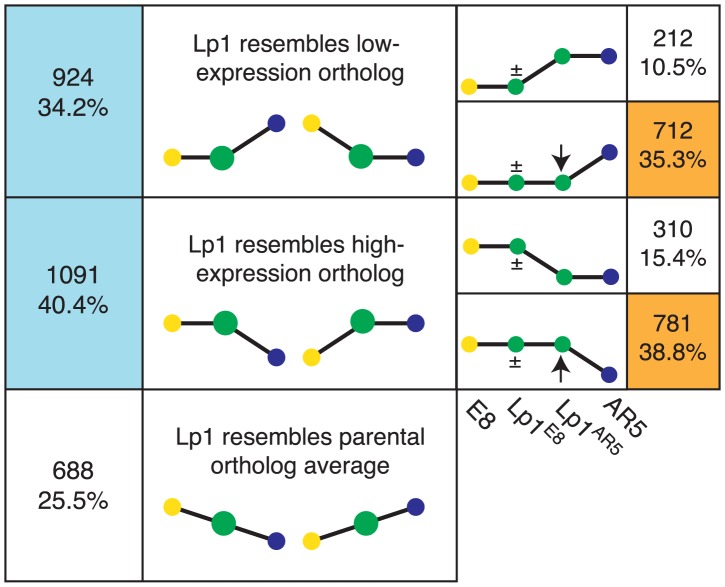
Frequent expression-level dominance from expression changes in the non-dominant homeolog. Genes showing differential expression in the parents were divided into those where total homeolog expression in Lp1 matches the lowly expressed parental ortholog, the highly expressed parental ortholog, or an intermediate level of expression (left panel; percentages are of all genes showing differential parental expression). Expression-level dominance patterns are indicated by blue boxes. On the right, expression-level dominance genes are divided into those where the non-dominant homeolog changed expression relative to the parents (orange boxes), and those where it did not (percentages are of all expression-level dominant genes). The heights of the yellow and blue dots represent expression of the parental orthologs. The green dot(s) represents expression of homeolog(s): larger green dots on the left represent both homeologs combined; on the right these have been separated into the two individual homeolog expression levels, as indicated below. The ± sign indicates the homeolog may or may not have changed expression. Expression-level dominance of only one parent is illustrated, but the numbers are for both.

Expression-level dominance can be achieved by various combinations of altered homeolog expression. In the one previous study where this was investigated, changes in the non-dominant homeolog were the driving force for most expression-level dominance [46]. To investigate the situation in Lp1, we took all genes that exhibited expression-level dominance and determined how the two homeologs contribute to this pattern (Figure 7). About three-quarters of genes showing expression level dominance involve a change in expression of the non-dominant homeolog. Therefore, the majority of genes that were differentially expressed in the parents have undergone a more complex set of transcriptional changes than just simple inheritance of parental gene expression patterns.

## Discussion

Here we investigated the fate of gene expression in an established, but relatively young (considerably less than 1 million year old), naturally occurring fungal allopolyploid. We developed a novel analytical pipeline that allowed us to utilize the power of high throughput mRNA sequencing to determine the expression levels of both homeologs in the allopolyploid on a genome-wide scale, and to compare these with the expression of the same genes in the closest known relatives to the parents. This analysis provides the first snapshot of global allopolyploid gene expression outside the plant kingdom, and illustrates a conservative transcriptome response to allopolyploidy, implying that genomic shock is largely buffered at the transcriptional level.

The strongest gene expression pattern we found was fewer differentially expressed genes in the allopolyploid than the parents, with more than two-thirds of all the genes we investigated not being differentially expressed in the allopolyploid. This loss of differential expression is the net result of two phenomena: a larger proportion of genes that lose differential expression following allopolyploidization (homeolog expression blending); and a smaller proportion of genes that gain it (homeolog expression bias). Therefore, at the global level, allopolyploidy is associated with conservative gene expression regulation, as has been observed previously [36], [39], [46]. Our analysis pipeline required us to remove around half the genes from the analysis, primarily because they were not (or barely) expressed in one or both parents, showed too little genetic variation to distinguish the two parental types, or were too short. This is likely to preferentially retain housekeeping genes and exclude genes that are expressed in response to specific environmental conditions. Given that housekeeping genes are likely to have predominantly conservative expression patterns, we may be over-estimating the proportion of genes that are not changing their expression levels in Lp1, and under-estimating those with significant changes in expression.

Our results are remarkably consistent with those of the pioneering RNA-seq study of cotton allopolyploids [46], despite these fungal and plant allopolyploids being different ages, of different ploidies, from different eukaryotic kingdoms, and having been analyzed with different algorithms/analytical methods. As outlined in Table 3, there are striking similarities in the proportions of genes in the major relative gene expression pattern outcomes we identified, including parental expression inheritance, homeolog expression blending, and homeolog expression bias. Additionally, the widespread expression-level dominance that we find, where combined total homeolog expression for a given gene in the allopolyploid is similar to that of one of the parental orthologs, is very similar to previously reported results [46] (Table 3). Also consistent with previous studies, we find that expression-level dominance is largely driven by transcriptional changes in the non-dominant homeolog, and that there is no particular bias towards dominance of the highly-expressed copy over the lowly-expressed copy [41], [46]. Cross-kingdom similarities in gross transcriptional change were previously noted [61], therefore we propose that the conserved transcriptional responses we observe are a general feature of allopolyploidy. Further RNA-seq studies from allopolyploids across the tree of life will be required to determine how general these allopolyploid transcriptional responses are.

We document only a small amount of gene loss in Lp1. Most losses involve the AR5 copy, contrary to the general trend of greater relative expression of AR5 homeologs within Lp1, suggesting that the E8-derived genome may have remained relatively inert. While our results do not address the mechanism behind gene loss, the proximity of several deletions to AT-rich regions implicate polyploidization-induced transposon activity in these deletions, as AT-rich regions are often associated with transposable elements in epichloë genomes [59]. Previous studies have shown that loss of duplicates is a routine phenomenon during and following polyploidy [30], and the low level of loss suggests that Lp1 is either a very young allopolyploid, or recalcitrant to gene loss (a property displayed by cotton allopolyploids [29]). Our current data do not allow us to resolve this question, as while we estimate the maximum time for allopolyploidization, we cannot provide a lower bound on its age. The strongest evidence that Lp1 is an established allopolyploid is the homogenization of the rDNA to a single parental type (Table S1 and Text S1), which is likely to have required a number of generations [62], [63].

**Figure 8:**
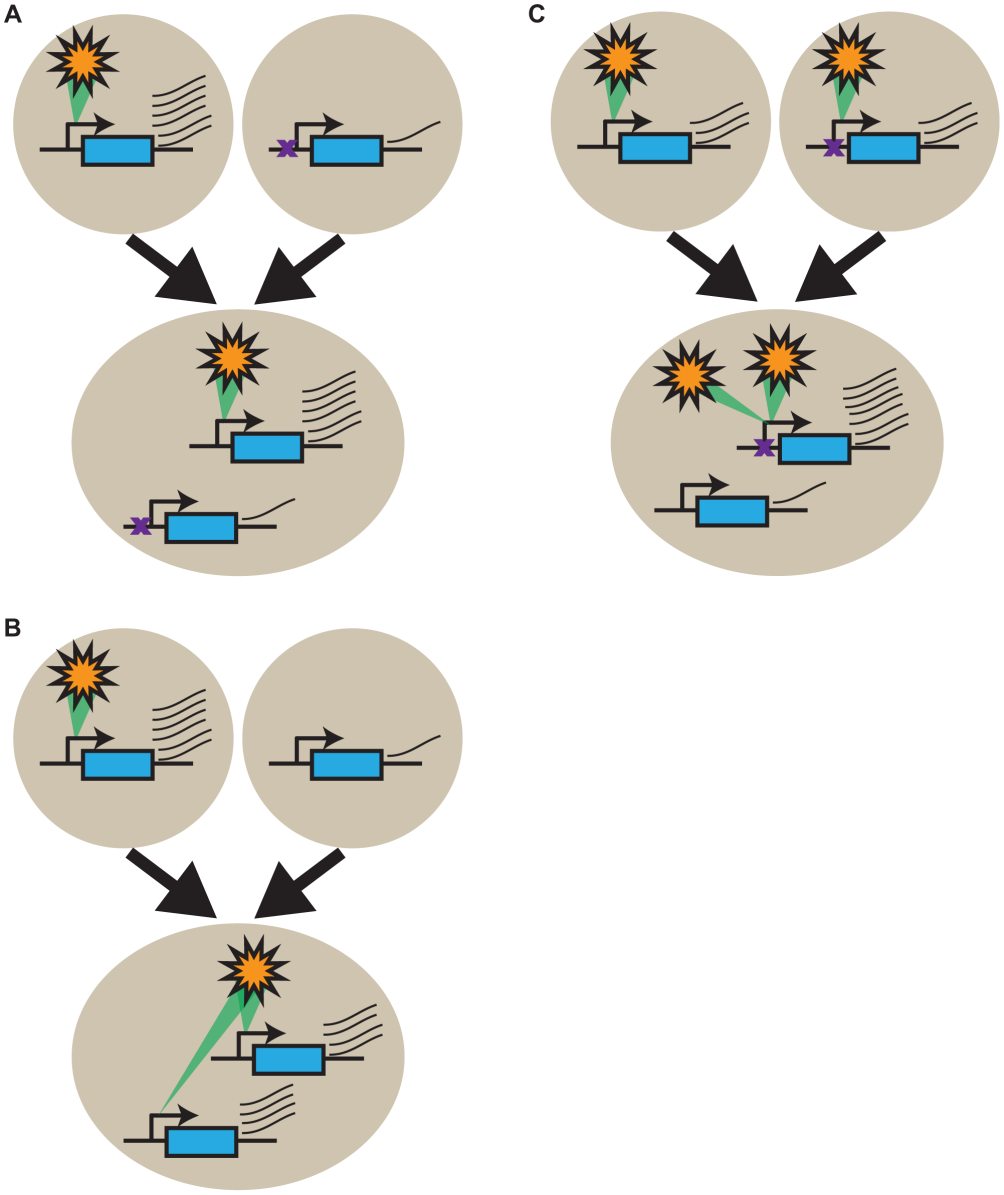
Intrinsic gene regulatory features underlie transcriptional response patterns in the allopolyploid. Change in expression of a gene (in blue) from the two parents (circles) to the allopolyploid (ellipse) is shown symbolically where a modulon (orange star; see text for details) positively regulates gene expression (green interaction). (A) Expression is high in one parent, with the modulon not active in the other. In the allopolyploid, this modulon only acts on its own homeolog, resulting in simple inheritance of biased gene expression. Failure of the modulon to recognize the other homeolog can result from a *cis*-acting change (purple cross) in one ortholog (B). The parental situation is the same as in (A), but the modulon acts on both homeologs, yielding homeolog expression blending in the allopolyploid. (C) Expression of the two orthologs is similar in the parents, but the modulon preferentially stimulates transcription of one homeolog in the allopolyploid, generating homeolog expression bias as a result of a *cis*-acting difference (purple cross) that does not change expression in the parent. Analogous outcomes will be achieved in all cases if the modulon is repressive rather than stimulatory (Figure S4). The promoter is indicated by a transcription arrow, and transcript levels by the number of thin wavy lines.

The GO-slim analyses we performed provided little support for selection having played a major role in shaping the expression patterns of most genes in Lp1. If these expression patterns are overwhelmingly not the result of strong gene-specific selective forces, what is causing them? We suggest that our results are primarily explained by intrinsic gene regulation factors [32], [64], [65], as originally proposed by Roose and Gottlieb [66]. Gene expression is the net outcome of a complex interplay of *cis*- and *trans*-acting factors [67], [68], including chromatin structure, transcription factors, effectors, RNAi, and nuclear position, that we collectively term a ‘modulon’ (Figure 8 and Figure S4). Changes anywhere in a modulon can impact gene expression, be it *cis*-acting changes (*e.g.* mutations in the promoter), epigenetic changes, and/or changes in the constellation of *trans*-acting factors. Expression differences between orthologs in the parents result from differences that were established in the modulon systems as the two parents evolved separately following speciation, while orthologs that are not differentially expressed are either governed by the same modulon system in both parents or coincidentally achieve the same expression through different modulon systems. Under this framework, the allopolyploid expression patterns we observe can be explained as follows: homeologs that faithfully inherit expression differences from their parents have modulon systems exhibiting little cross talk between the homeologs (Figure 8A). Conversely, genes that show homeolog expression blending result from modulon systems that partially or fully cross talk between homeologs (Figure 8B). Finally, genes that gain expression bias following allopolyploidy result from modulon systems that preferentially regulate one homeolog over the other, resembling classical dominance (Figure 8C). Therefore we propose that the allopolyploid gene expression patterns we observe are predominantly the net outcome of the modulon features that existed in the parents, although it will be important to distinguish the effects of genome doubling [69].

If these patterns of allopolyploid expression fate are predominantly the result of regulatory differences present in the parents, they open a window into the evolution of gene regulation following speciation [61], [66]. Genes where the homeologs have faithfully inherited expression differences from the parents have developed full transcriptional independence in the time since the two parents speciated, whereas those showing homeolog expression blending have not evolved independence since speciation. We can use the results from Figure 6 to estimate the proportions of genes that have developed independence, partial independence and have not developed independence via the number of genes that show simple inheritance of biased expression, homeolog expression bias, and homeolog expression blending, respectively. Depending on how we treat genes with non-biased expression in both the parents and the allopolyploid (see ), our results suggest that 13–28% of genes have evolved independent regulation, 19% have evolved partial independence, and 53–68% have not evolved independence. The results presented in Figure 7 can also be used to estimate these proportions, as the first and third rows represent independent regulation, and the second and fourth rows represent partial and no independent regulation (combined). These totals (25.9% and 74.1% respectively) both fall within the ranges estimated from Figure 6.

We are proposing that the transcriptional responses to allopolyploidy are largely a passive outcome of regulatory evolution that has occurred in the parents following speciation. In principle, they could also be explained by more saltational events that are specific to the allopolyploid, such as genome-wide chromatin reprograming [70]. However, we believe that the striking cross-kingdom conservation in transcriptional responses supports the view that the majority of these responses are outcomes of parental regulatory evolution. It is not clear why saltaic mechanisms should result in conserved patterns of transcriptional responses between species with highly diverged genome structures, global expression regulators, and chromatin networks. However, if these conserved gene expression response patterns largely result from mutations accumulating in modulons over time, then a roughly monotonic increase in the proportions of genes that evolve partial and full transcriptional independence would be expected (Figure S5). One prediction of this hypothesis is that the more genetically diverged the parents of two allopolyploids are, the more different the allopolyploid transcriptional responses will be. Clearly more allopolyploid transcriptome studies are required to determine whether this is true and how conserved the transcriptional responses are. It will also be of great interest to determine whether similar genes evolve similar regulatory patterns in different allopolyploids, and how the effects of homeolog interference influence the transcriptional response [71].

In conclusion, we present the first study of global gene expression in a fungal allopolyploid species. We show that most genes are still retained in duplicate, suggesting that Lp1 is either a young allopolyploid or is resistant to the gene loss process that often accompanies allopolyploidization. We find a mixture of expression patterns, with the homeologs for many genes retaining the gene expression patterns seen in the parents, fewer showing less biased expression than seen in the parents, and fewer still developing biased expression. Strikingly, these expression patterns are remarkably concordant with those recently ascertained for allopolyploid cotton, suggesting there exists a general pattern of interspecific allopolyploid gene expression fate that is largely independent of taxonomic kingdom, gene repertoire or local environment. We conclude that the transcriptional response to allopolyploidy is conservative and conserved, reflecting the stochastic nature of genetic regulatory evolution. Our work suggests that the fate of allopolyploid gene expression follows general principles that apply across eukaryotes, and that allopolyploid transcriptomes are a novel and powerful way to unmask the regulatory changes that evolve following speciation.

## Materials and Methods

### Strains and growth conditions

Three filamentous fungi from the ascomycete family Clavicipitaceae were used in this study: the asexual, diploid interspecies fungal allopolyploid *Neotyphodium lolii*×*Epichloë typhina* Lp1 (*syn.* AR6) [50], [72], the haploid asexual species *Neotyphodium lolii* AR5, and the haploid sexual species *Epichloë typhina* E8. Cultures were grown in 2.4% potato dextrose (PD) media until maturity, washed twice with double distilled water, then resuspended in a defined medium (CDGN) comprised of Czapek Dox salts [73] containing 100 mM glucose and 10 mM ammonium sulphate.

### RNA isolation and high throughput sequencing

The fungal cultures were filtered and washed, and ∼100 mg of mycelium (wet weight) per sample was used for RNA extraction, which was performed using an RNeasy Plant RNA extraction kit (Qiagen) according to the manufacturer's instructions. Total RNA samples were treated with DNAse I to remove contaminant DNA, mRNA was extracted using polyA selection, and Illumina sequencing libraries were prepared using standard protocols. Libraries were sequenced on the HiSeq 2000 – 100 bp single end sequences for AR5 and E8, and 100 bp paired end sequences for Lp1. Two biological replicates were sequenced for each. Bases were called using CASAVA (v. 1.7.0, Illumina, Hayward, CA, USA). Sequencing quality control was performed using the SolexaQA package (v. 1.10) [74]. In all six datasets, >79% of bases were sequenced to Q30 or higher (*i.e.*, bases have a probability of error, *P*<0.001).

### Transcriptome sequencing data analysis methods

Full details of the analyses that were performed to process the sequences, to create the reference gene sets that allowed us to bin Lp1 reads as coming from the AR5-like and E8-like homeologs, and to perform these allocations, are described in Figure S1, Figure S6 and Protocol S1. Briefly, two E8 and AR5-like reference gene sets were created by modifying an existing annotated set of gene models from the closely related *Epichloë festucae* E2368 using SNPs that were generated from the parental and Lp1 transcriptome data. Lp1 reads were then mapped with high stringency to the informative sites of these two reference gene sets to determine from which parental copy each Lp1 sequence read derived. Genes that had fewer than five reads in one or both of the parental transcriptomes were excluded from both E8 and AR5 reference gene sets, as were genes where the informative regions were less than 150 bp.

### Statistics

The statistical significance of gene expression differences between AR5-like and E8-like homeologs in the Lp1 allopolyploid data was determined using Fisher's Exact Test [75] as implemented in the R [76] package DEGseq v.1.8.0 [77]. A correction for multiple testing was applied using the False Discovery Rate (FDR) approach described by Storey and Tibshirani [78]. The fold difference for each gene *i* was calculated as
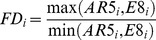
(1)where gene counts were converted to ‘reads per million’ to normalize for unequal numbers of reads between biological replicates, as well as unequal numbers of reads mapping to the AR5-like and E8-like homeolog references. Subsequent analyses were performed in Excel. Differential gene expression was defined throughout this work as statistical significance and a 2-fold or greater difference in normalized gene counts, except where otherwise noted.

### Gene ontology

Gene ontology analysis was performed as described in Text S2.

### PCR-RFLP

RFLPs were identified in candidate genes using the read data in IGV [79], and primers were designed to flank them (Table S2). Genomic DNA was extracted, using the protocol of Peintner and colleagues [80], from the same material used to make the RNA. End-point PCR was performed using standard procedures with Ex-Taq polymerase (Takara). PCR products were precipitated with isopropanol and then digested with the appropriate restriction enzyme according to manufacturers' instructions. The products were run on 2% agarose or 8% native polyacrylamide gels.

### Dating

Approximate estimates of lineage ages were made using a mutation rate of 1×10^−9^/site/year [81] and a cumulative haploid sequence length of 7,123,190 bp in the final masked AR5-like and E8-like homeolog references. See Text S1, Figure S6 and Figure S7 for details.

## Supporting Information

Figure S1 Approach to determine homeolog-specific gene expression. Methods differ slightly for the haploid parental species AR5 and E8 compared to the diploid allopolyploid species Lp1. Dotted lines indicate steps where the same operations were performed for all three species. For detailed descriptions, see Protocol S1.
(PDF)

Figure S2 PCR-RFLP of non-clustered extreme differentially expressed genes in Lp1 and the parents. Three genes showing extreme differential expression but no clustering are shown. Diagram is as in Figure 4, except that 2% agarose gels were used.
(PDF)

Figure S3 GO-slim analysis of sets of genes with various expression patterns in Lp1. The graph at the top of each gene set shows the number of genes present in each GO-slim category for all mapped genes (blue), and the subset of genes under investigation (orange). Categories in the grey box have no genes in the subset of genes. The graph below shows the fold over-representation (red) and under-representation (pale blue) of the subset of genes relative to all genes. Only categories that differ between the subset of genes and all genes by at least 1.5-fold are shown. Green stars represent categories that show consistent behavior in at least two different gene subsets (see Text S2 for details). The gene subsets are: (A) Genes with no change in expression. (B) Homeolog expression blending genes. (C) Homeolog expression bias genes. (D) Homeolog expression reversal genes. (E) All differentially expressed genes in Lp1.
(PDF)

Figure S4 Model for changes in homeolog expression following allopolyploidy as a result of repressive modulon regulatory systems. Diagram is the same as Figure 8, except that the modulons depicted (orange stars with red interactions) regulate genes by repressing gene transcription, rather than activating it. (A) Simple inheritance of biased gene expression. (B) Homeolog expression blending in the allopolyploid. (C) Homeolog expression bias.
(PDF)

Figure S5 Evolutionary trajectory of gene regulation. The evolution of independence in gene regulatory systems in two species following the speciation event that separated them is represented. Initially genes have identical modulons, so none have any independence. However, changes anywhere in the modulon system for each gene may result in that gene becoming more independent in its transcriptional regulation. We propose that, stochastically, the development of partial and full independence occur at similar rates in different organisms, thus accounting for the conserved gene expression responses seen in Lp1 and cotton following allopolyploidy.
(PDF)

Figure S6 Schematic diagram of phylogenetic relationships among the strains used in this study. The reference strain, E2368 is a close relative of AR5. The green branch indicates ‘ancestral’ SNPs shared by AR5, E8 and Lp1 relative to the reference strain, E2368. Red branches indicate SNPs that are unique to either AR5 or E8 (‘AR5-unique’ and ‘E8-unique’, respectively). Blue branches indicate SNPs that are unique to Lp1 (‘Lp1-unique’), some of which can be classified as falling on the AR5-like lineage (‘Lp1-AR5’) or the E8-like lineage (‘Lp1-E8’). The number of SNPs on the shortest parental branch, here the AR5 parental lineage (‘AR5-unique’), provides an upper bound on the allopolyploidization time. It is not possible to place a lower bound on this event with available data.
(PDF)

Figure S7 Diagnostic markers of mitochondrial DNA ancestry in Lp1. There are diagnostic substitutions at nucleotide positions 29443, 39542 and 54076 in the mitochondrial genome that distinguish AR5 and E8. Grey horizontal bars indicate independent sequence reads, and the red bases indicate the AR5 variant at the diagnostic positions. At all three diagnostic positions, all Lp1 sequence reads carry the AR5 nucleotide variant, thus suggesting that mitochondrial genomes from the AR5 parent completely replaced their E8 counterparts in Lp1.
(PDF)

Protocol S1 Detailed analysis materials and methods. Descriptions of the methods used for base-calling of Illumina reads, the E2368 gene models used, calling of SNPs from Lp1 and the parents, construction of the transcriptome references and how these were masked, how reads from the allopolyploid were allocated to the two parental classes, and how the reads were visualized are all presented.
(DOCX)

Table S1 Lp1 contains E8 but not AR5 rDNA sequences. Table showing counts of reads from the parents and Lp1 that were mapped to parental ITS reference sequences.
(DOCX)

Table S2 PCR primers used in this study.
(DOCX)

Text S1 Dating the allopolyploidization event. Description of how the age range of Lp1 was estimated.
(DOCX)

Text S2 GO-slim analysis. Description of how the different gene sets were analyzed using GO-slim to look for evidence of selection having shaped the transcriptional response to allopolyploidy.
(DOCX)

Text S3 Determining the proportion of genes that have evolved varying degrees of transcriptional independence since speciation of the parents. Description of the logic and method used to calculate the percent range of genes that have undergone full, partial and no transcriptional independence since the speciation of the two parent species prior to the allopolyploidization event.
(DOCX)
