# Supplementary figures and images for: An Interspecific Fungal Hybrid Reveals Cross-Kingdom Rules for Allopolyploid Gene Expression Patterns
Source: PLoS Genet. 2014 Mar 6;10(3):e1004180. doi: 10.1371/journal.pgen.1004180 (PMC3945203; doi:10.1371/journal.pgen.1004180)

**AR5**

**Lp1**

**E8**

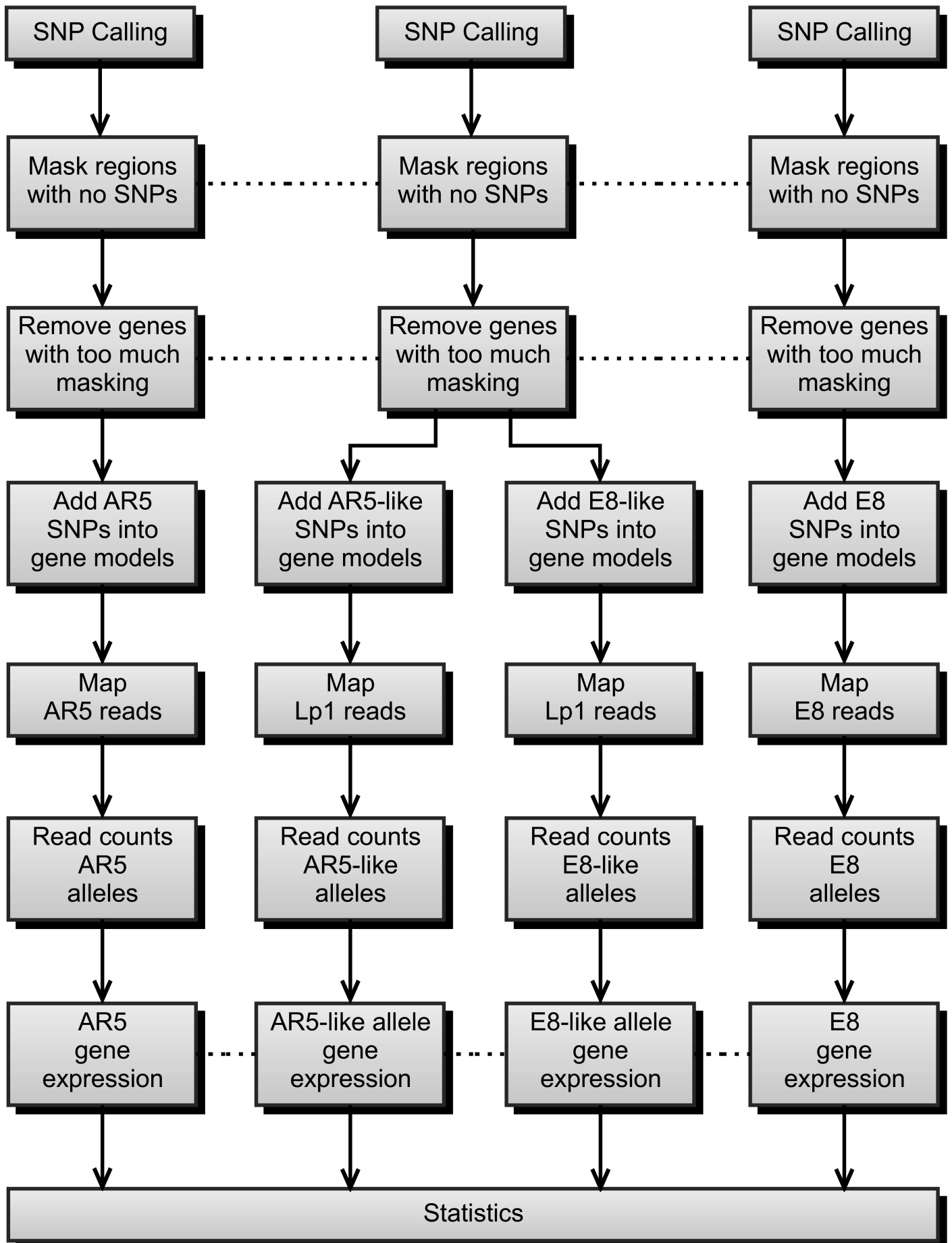

Supplement: Figure S1 — Approach to determine homeolog-specific gene expression. Methods differ slightly for the haploid parental species AR5 and E8 compared to the diploid allopolyploid species Lp1. Dotted lines indicate steps where the same operations were performed for all three species. For detailed descriptions, see Protocol S1. (PDF) [file pgen.1004180.s001.pdf]

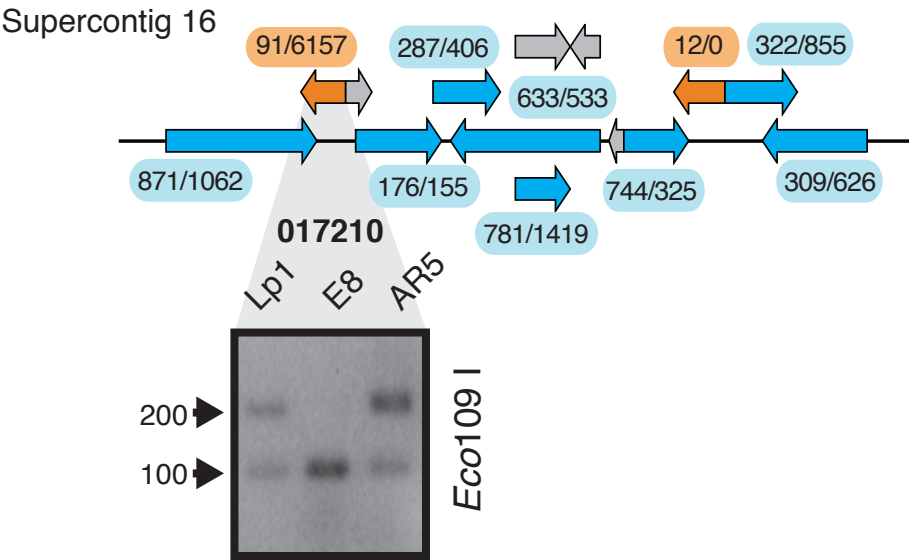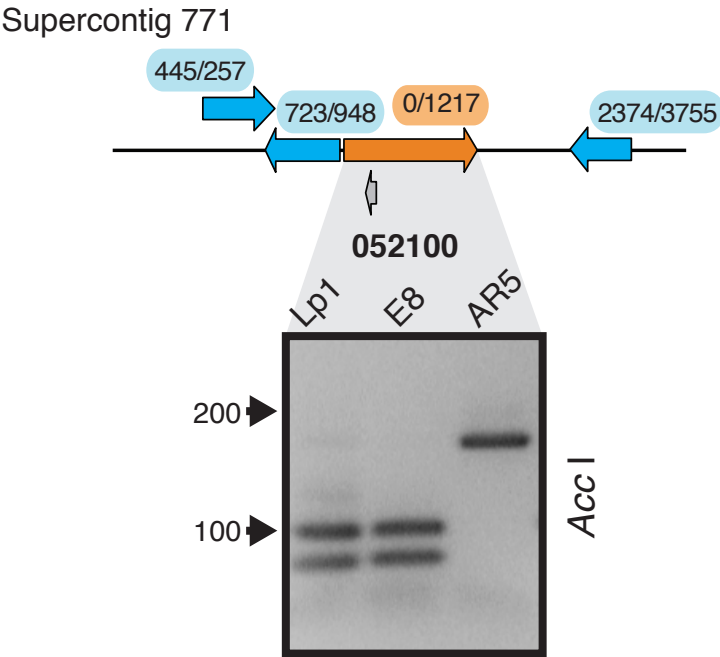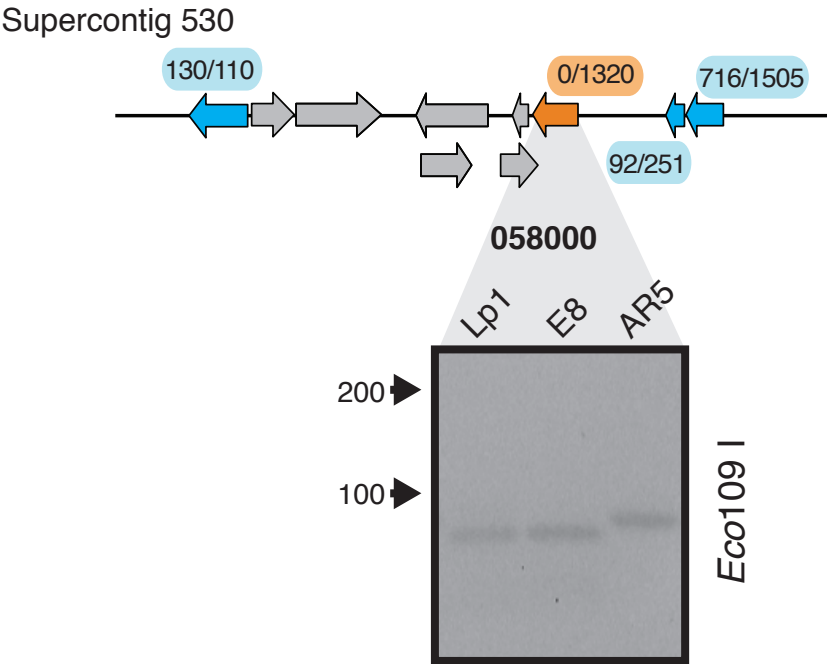

Supplement: Figure S2 — PCR-RFLP of non-clustered extreme differentially expressed genes in Lp1 and the parents. Three genes showing extreme differential expression but no clustering are shown. Diagram is as in Figure 4, except that 2% agarose gels were used. (PDF) [file pgen.1004180.s002.pdf]

A

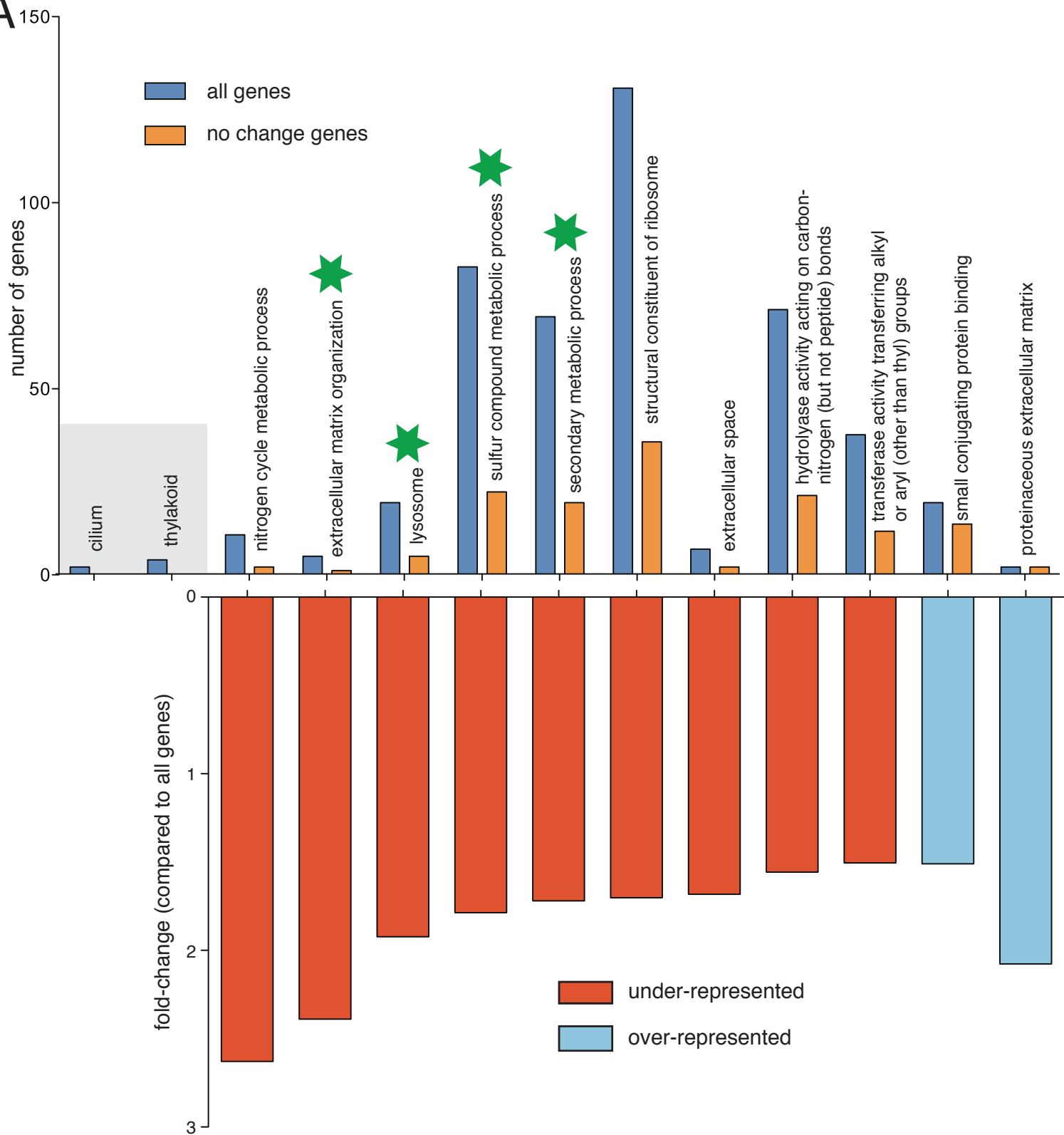

B

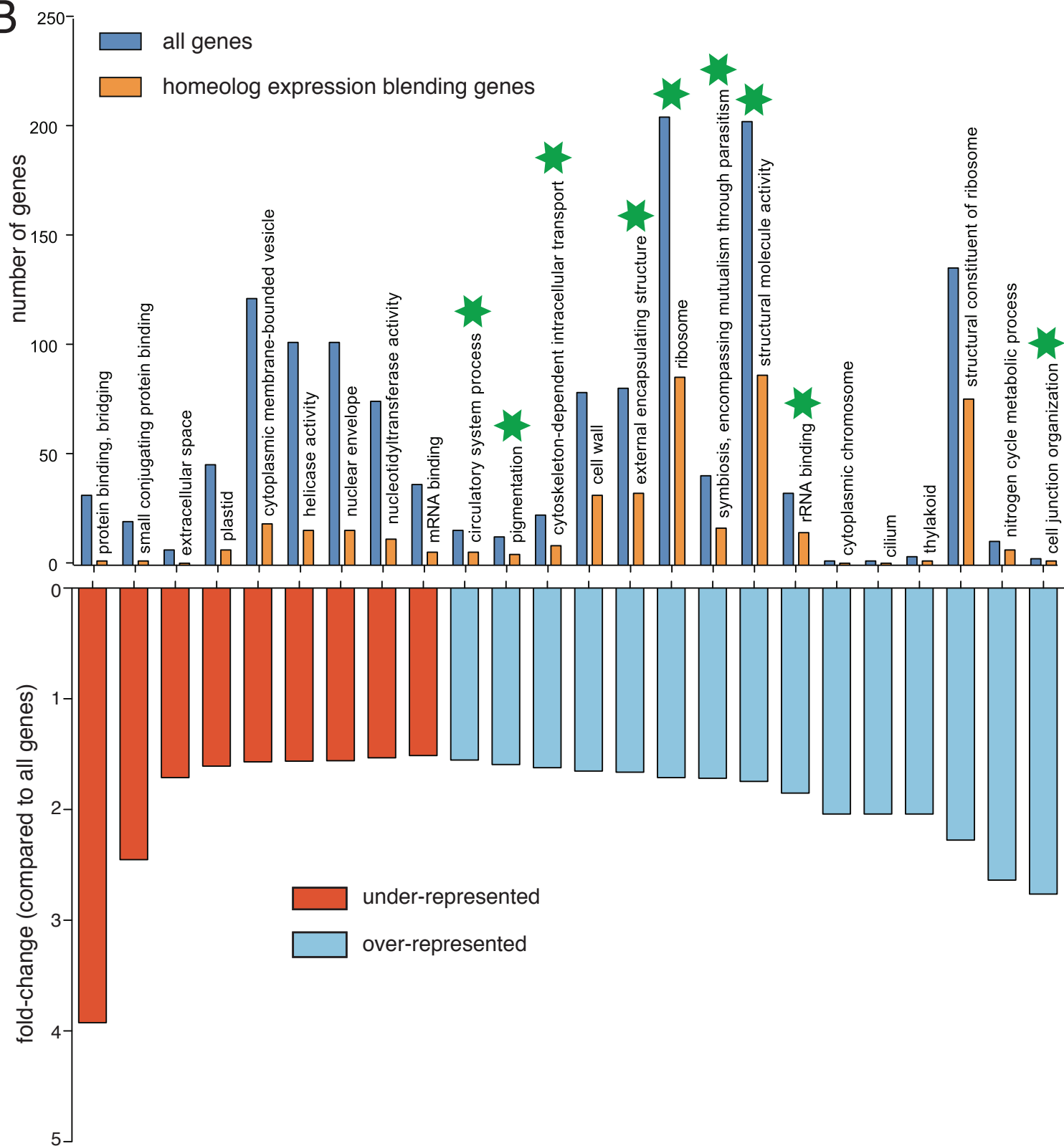

C

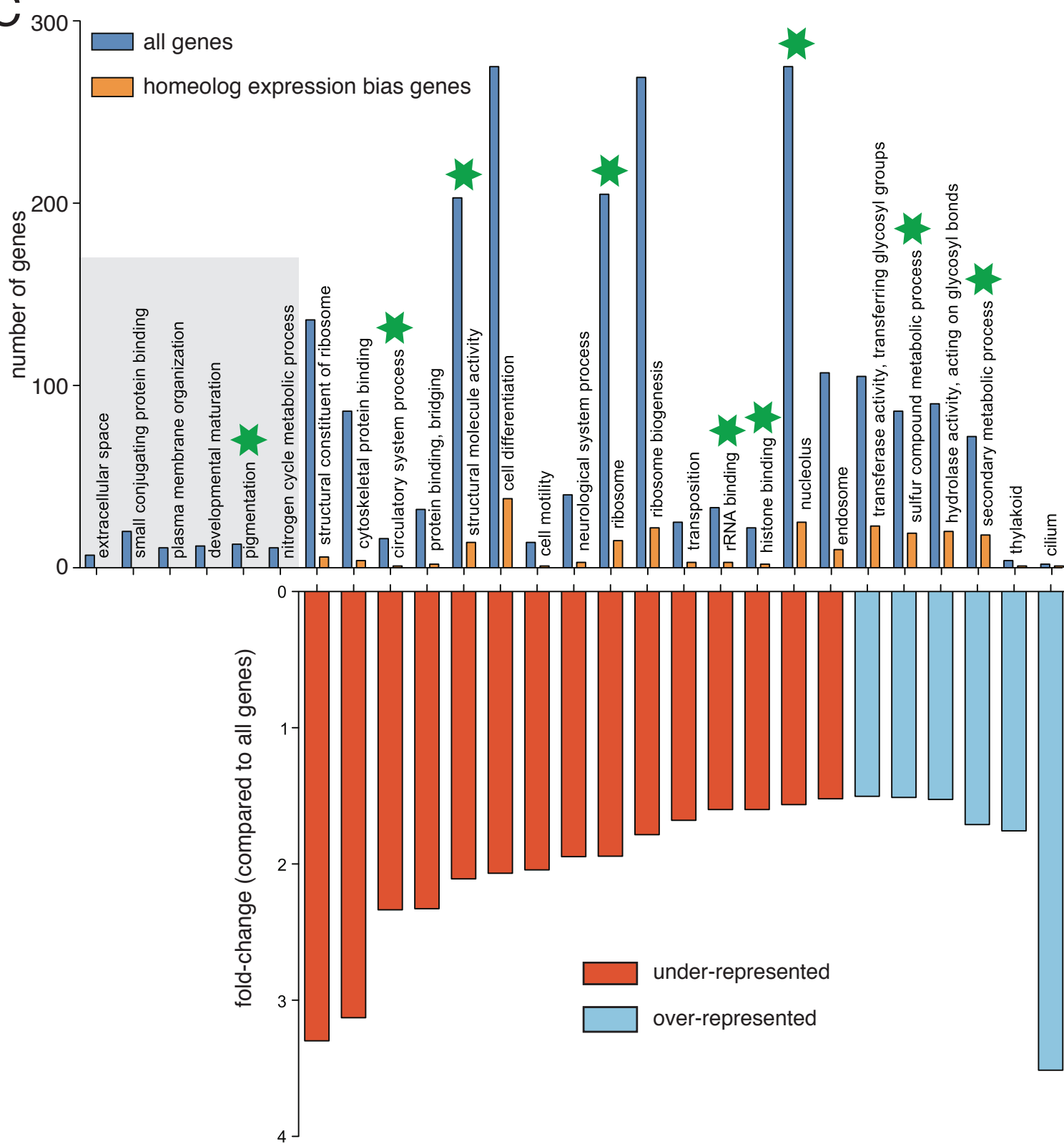

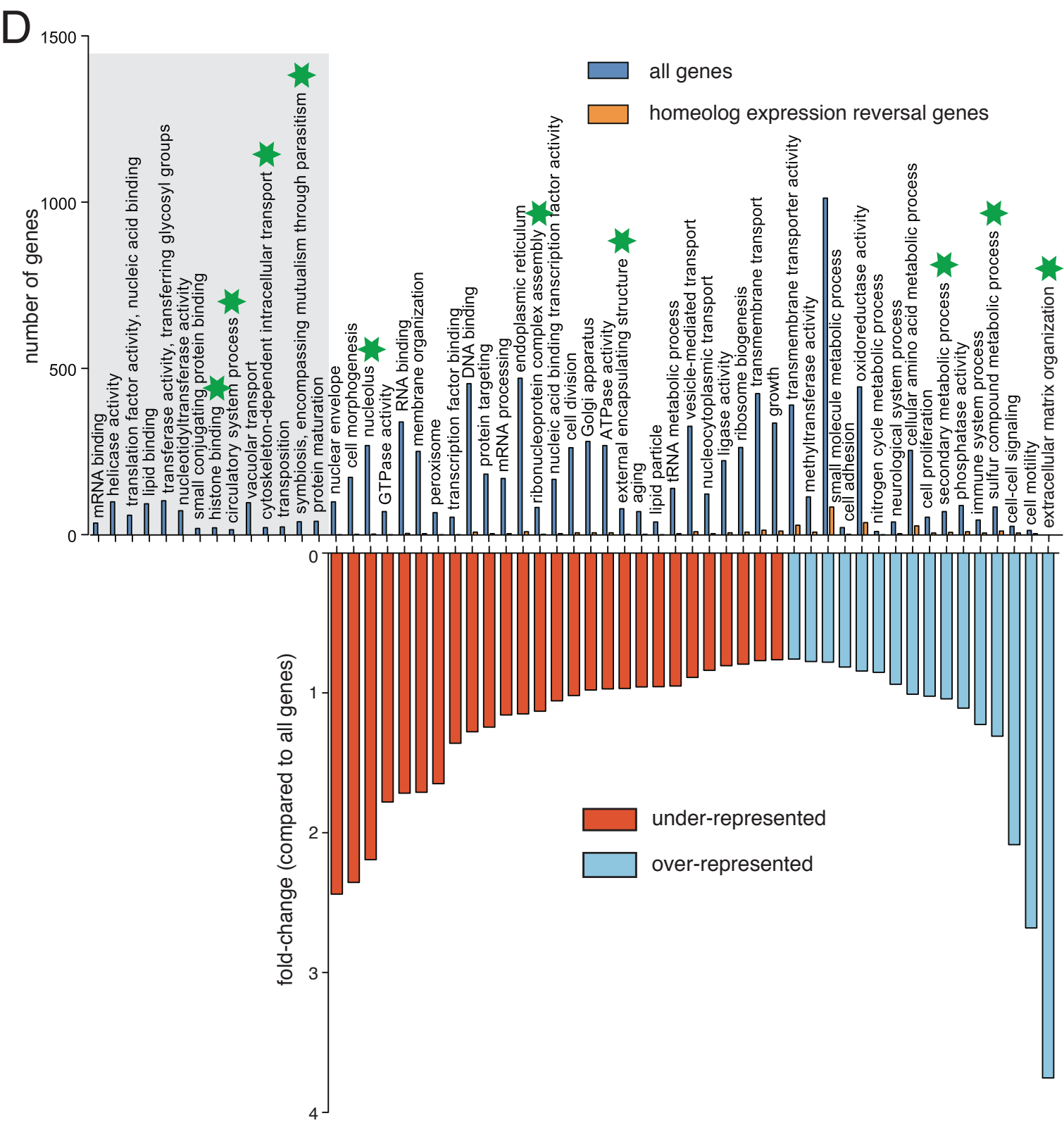

11

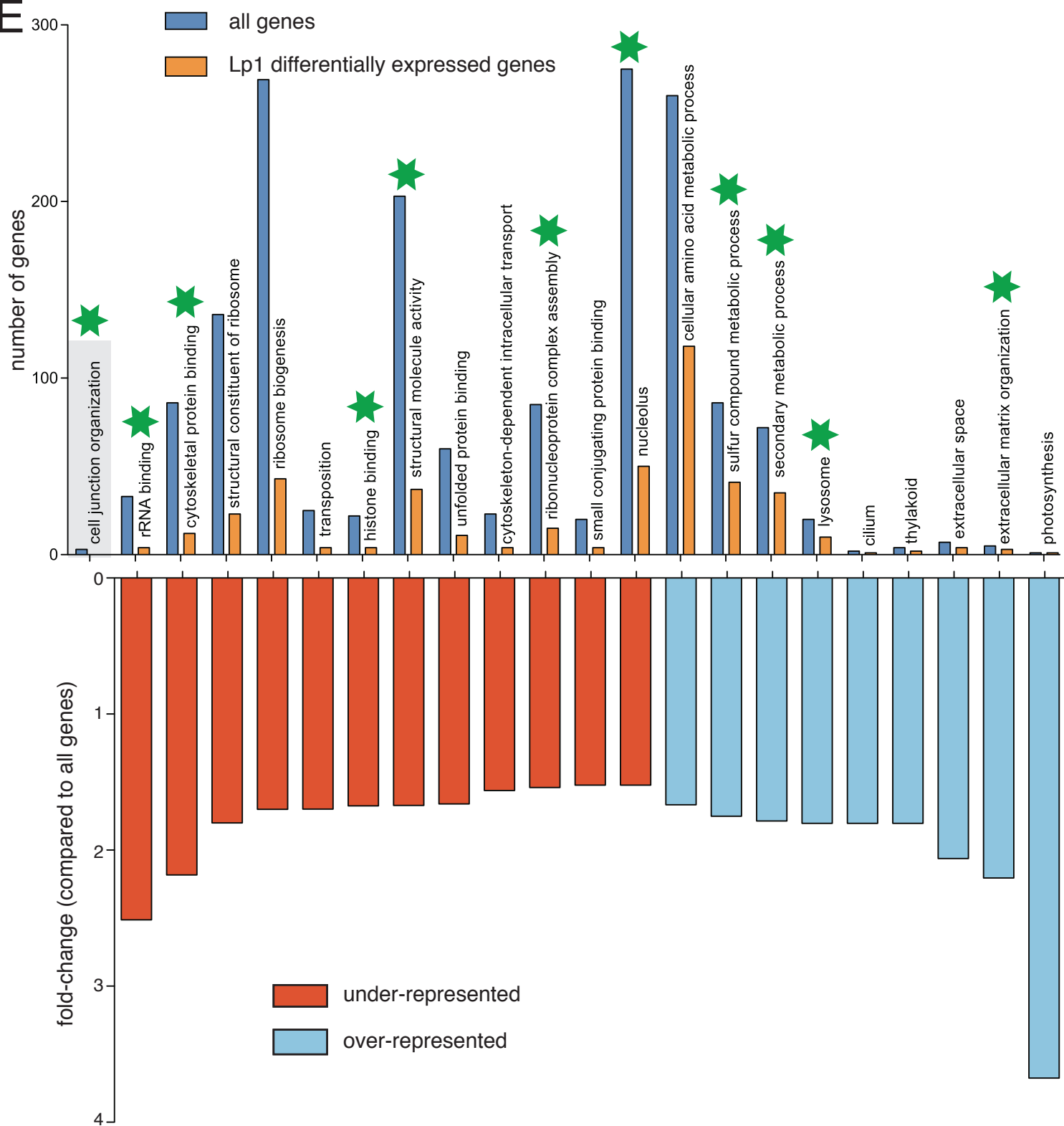

Supplement: Figure S3 — GO-slim analysis of sets of genes with various expression patterns in Lp1. The graph at the top of each gene set shows the number of genes present in each GO-slim category for all mapped genes (blue), and the subset of genes under investigation (orange). Categories in the grey box have no genes in the subset of genes. The graph below shows the fold over-representation (red) and under-representation (pale blue) of the subset of genes relative to all genes. Only categories that differ between the subset of genes and all genes by at least 1.5-fold are shown. Green stars represent categories that show consistent behavior in at least two different gene subsets (see Text S2 for details). The gene subsets are: (A) Genes with no change in expression. (B) Homeolog expression blending genes. (C) Homeolog expression bias genes. (D) Homeolog expression reversal genes. (E) All differentially expressed genes in Lp1. (PDF) [file pgen.1004180.s003.pdf]

A

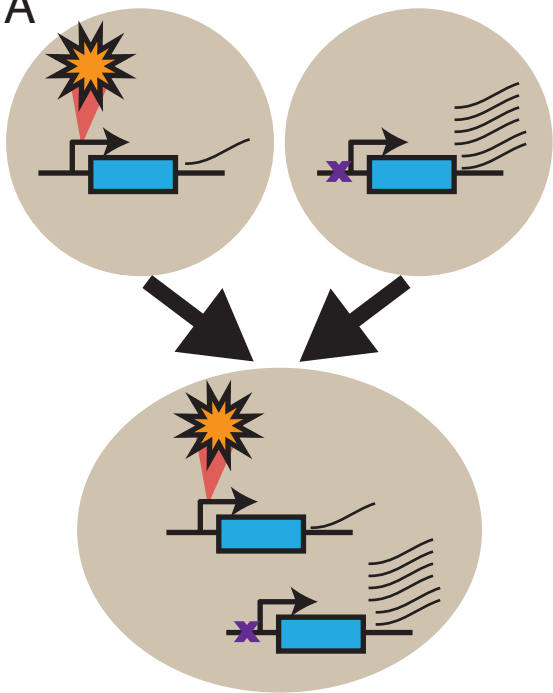

B

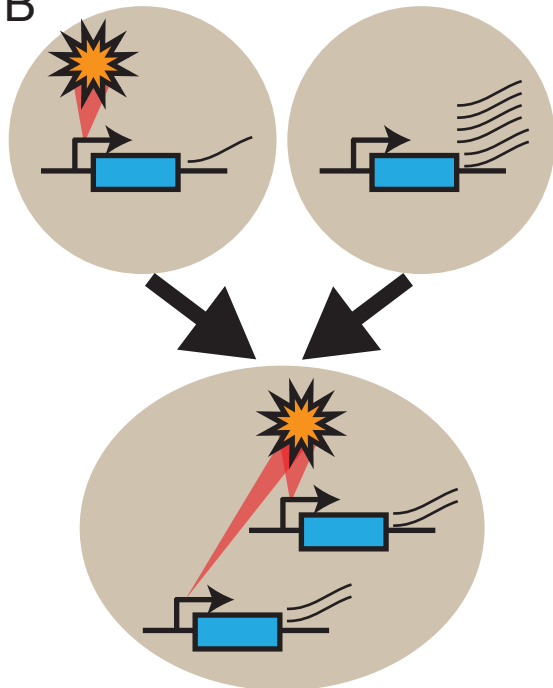

C

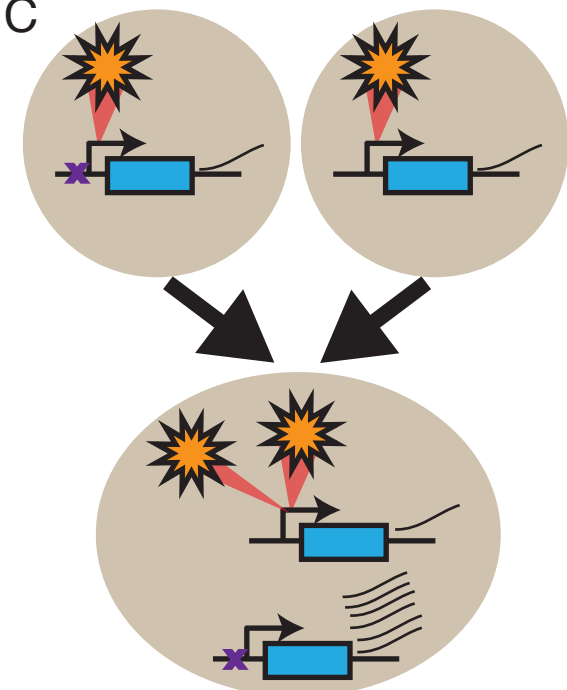

Supplement: Figure S4 — Model for changes in homeolog expression following allopolyploidy as a result of repressive modulon regulatory systems. Diagram is the same as Figure 8, except that the modulons depicted (orange stars with red interactions) regulate genes by repressing gene transcription, rather than activating it. (A) Simple inheritance of biased gene expression. (B) Homeolog expression blending in the allopolyploid. (C) Homeolog expression bias. (PDF) [file pgen.1004180.s004.pdf]

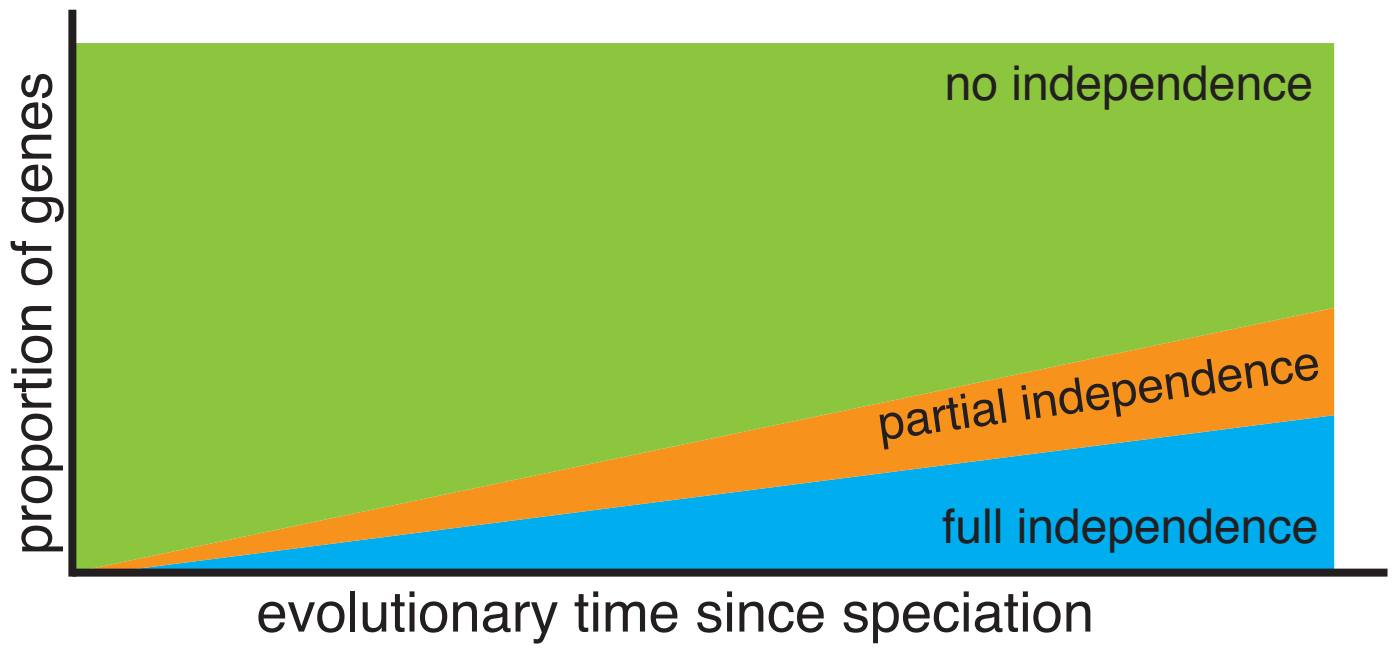

Supplement: Figure S5 — Evolutionary trajectory of gene regulation. The evolution of independence in gene regulatory systems in two species following the speciation event that separated them is represented. Initially genes have identical modulons, so none have any independence. However, changes anywhere in the modulon system for each gene may result in that gene becoming more independent in its transcriptional regulation. We propose that, stochastically, the development of partial and full independence occur at similar rates in different organisms, thus accounting for the conserved gene expression responses seen in Lp1 and cotton following allopolyploidy. (PDF) [file pgen.1004180.s005.pdf]

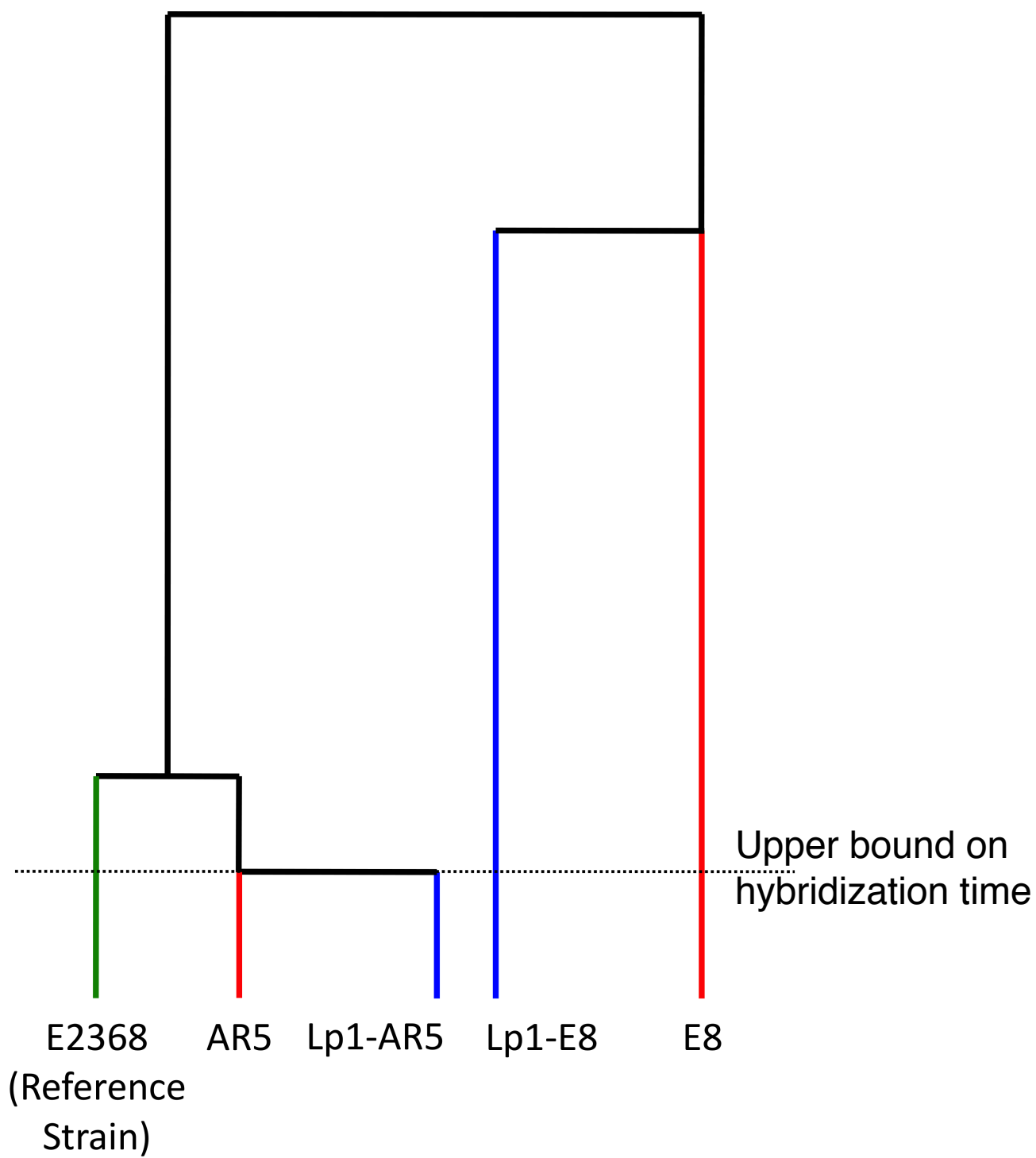

Supplement: Figure S6 — Schematic diagram of phylogenetic relationships among the strains used in this study. The reference strain, E2368 is a close relative of AR5. The green branch indicates ‘ancestral’ SNPs shared by AR5, E8 and Lp1 relative to the reference strain, E2368. Red branches indicate SNPs that are unique to either AR5 or E8 (‘AR5-unique’ and ‘E8-unique’, respectively). Blue branches indicate SNPs that are unique to Lp1 (‘Lp1-unique’), some of which can be classified as falling on the AR5-like lineage (‘Lp1-AR5’) or the E8-like lineage (‘Lp1-E8’). The number of SNPs on the shortest parental branch, here the AR5 parental lineage (‘AR5-unique’), provides an upper bound on the allopolyploidization time. It is not possible to place a lower bound on this event with available data. (PDF) [file pgen.1004180.s006.pdf]

29443

39542

54076

AR5

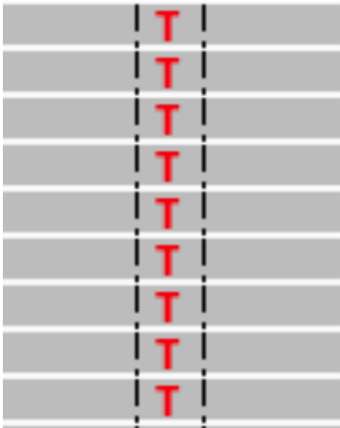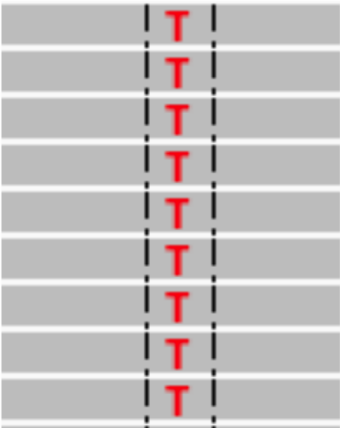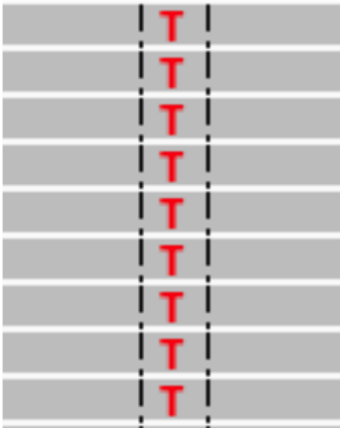

E8

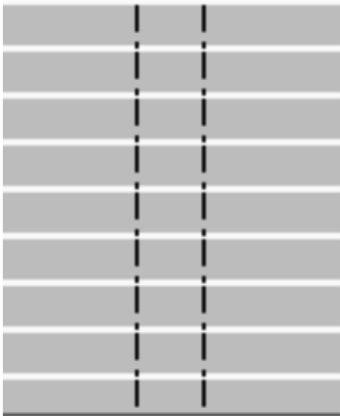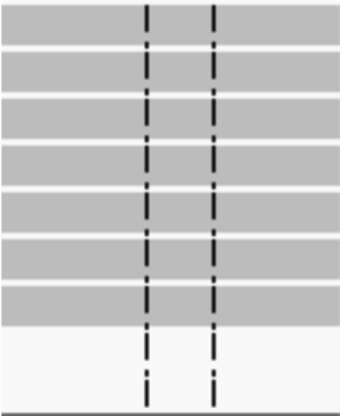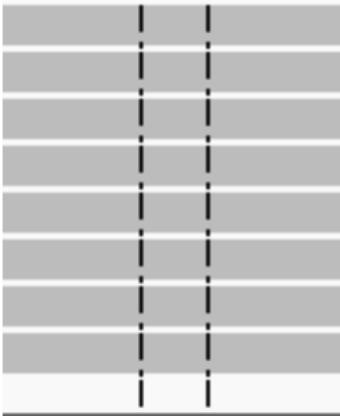

Lp1

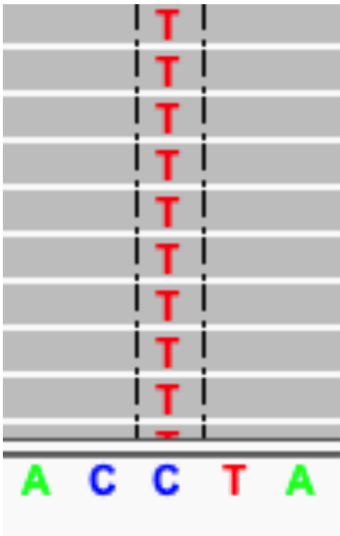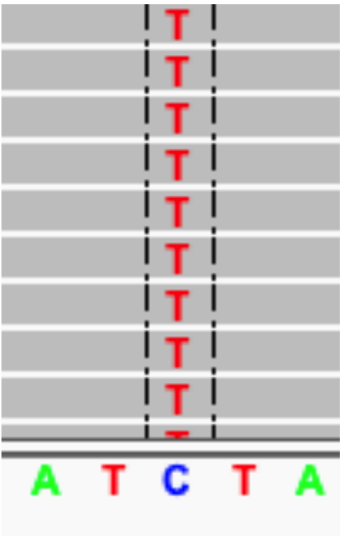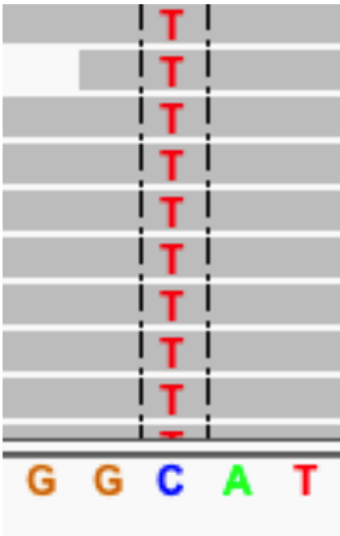

Supplement: Figure S7 — Diagnostic markers of mitochondrial DNA ancestry in Lp1. There are diagnostic substitutions at nucleotide positions 29443, 39542 and 54076 in the mitochondrial genome that distinguish AR5 and E8. Grey horizontal bars indicate independent sequence reads, and the red bases indicate the AR5 variant at the diagnostic positions. At all three diagnostic positions, all Lp1 sequence reads carry the AR5 nucleotide variant, thus suggesting that mitochondrial genomes from the AR5 parent completely replaced their E8 counterparts in Lp1. (PDF) [file pgen.1004180.s007.pdf]
