# Supplementary material for: An Interspecific Fungal Hybrid Reveals Cross-Kingdom Rules for Allopolyploid Gene Expression Patterns
Source: PLoS Genet. 2014 Mar 6;10(3):e1004180. doi: 10.1371/journal.pgen.1004180 (PMC3945203; doi:10.1371/journal.pgen.1004180)
Supplement: Protocol S1 — Detailed analysis materials and methods. Descriptions of the methods used for base-calling of Illumina reads, the E2368 gene models used, calling of SNPs from Lp1 and the parents, construction of the transcriptome references and how these were masked, how reads from the allopolyploid were allocated to the two parental classes, and how the reads were visualized are all presented. (DOCX) [file pgen.1004180.s008.docx]

**Protocol S1**

Base-calling

Bases were called using CASAVA (v. 1.7.0, Illumina, Hayward, CA, USA). Sequencing quality control was performed using the SolexaQA package (v. 1.10) [74]. In all six datasets, >79% of bases were sequenced to Q30 or higher (i.e., bases have a probability of error, *P* < 0.001).

Gene models

An annotated genome sequence for the closely related *Epichloë festucae* E2368 (http://www.endophyte.uky.edu) was modified to create two reference gene sets for transcriptome mapping. Gene models (EfM2; *n* = 12,199) were preprocessed to remove contaminant Soybean (*Glycine max*) sequences (*n* = 7) and alternative splicing variants (*n* = 997). To limit reads cross-mapping between closely related genes, the gene models were blasted recursively (*blastn*, BLAST+ v. 2.2.23 [83]) to exclude all but one representative of each gene set in which members match with ≥90% nucleotide identity over ≥50% of the query sequence length with an E-value ≤0.05 (*n* = 1,359). Following this step, the reference gene set contained 9,836 genes (80.6%).

SNP calling

Reads from Lp1, AR5 and E8 were trimmed dynamically using the *LengthSort* module of SolexaQA v. 1.10 [74], which returned the longest contiguous sequence containing bases with a miscall error rate ≤0.05. Reads ≥50-bp were mapped to the cleaned gene references using the Burrows-Wheeler transform aligner BWA v. 0.5.9-r16 [84]. Only reads that mapped uniquely were accepted, and an alignment of these reads to the reference gene sequences was converted to base calls at every position in the reference gene set using the *pileup* module of Samtools v. 0.1.7 [85]. SNPs were called if a mismatch was detected in ≥5 independent reads, and the position had sufficient read coverage (≥5 independent reads) across all three datasets (Lp1, AR5 and E8).

SNPs were further classified by their relative distribution among strains: i) common to Lp1, AR5 and E8 (‘ancestral’); ii) shared between Lp1 and AR5 (‘AR5-shared’); iii) shared between Lp1 and E8 (‘E8-shared’); iv) unique to Lp1 (‘Lp1-unique’); v) unique to AR5 (‘AR5-unique’); and vi) unique to E8 (‘E8-unique’). Classes ii and iii represent SNPs distinguishing the two homeologs in the allopolyploid, and therefore provide the power to resolve the parental origin of allopolyploid transcripts.

A set of new SNPs that are unique to Lp1 were identified by mapping the Lp1 reads to the two reference gene sets with low stringency. Two temporary gene references were created by introducing SNPs into the reference gene set. Both references contained the ancestral and Lp1-unique SNPs, but differed in carrying either the AR5-shared or E8-shared SNPs. Trimmed Lp1 reads were mapped to these two gene references with high stringency using BWA. Only reads that mapped uniquely with no mismatches were accepted, and SNPs were called on this subset of reads as described above. Because only perfect read matches were allowed, this process determined linkage between some of the Lp1-unique SNPs and SNPs with known AR5 or E8 ancestry. Lp1-unique SNPs were therefore further defined as being located on the AR5 lineage (‘Lp1-AR5’), the E8 lineage (‘Lp1-E8’), or placed in an ‘unknown’ category (‘Lp1-unclassified’). These subsets of SNPs are described in **Table 2**.

Transcriptome reference

Two reference gene sets were created for transcriptome mapping by introducing the SNPs identified above into the E2368 gene models. The AR5 reference contained the ancestral, AR5-shared and Lp1-AR5 SNPs, while the E8 reference contained the ancestral, E8-shared and Lp1-E8 SNPs.

Reference masking

SNPs could not be called at nucleotide positions with insufficient sequencing coverage (<5 independent reads) in the AR5, E8 or Lp1 datasets, therefore the reference sequences were masked at these positions. Further, to prevent mapping of uninformative reads (*i.e.*, reads that do not overlap a known SNP), nucleotides were masked if they occur ≥25-bp away from at least one AR5-shared, E8-shared, Lp1-AR5 or Lp1-E8 SNP. To keep the mapping process comparable, the same nucleotides were masked in both gene references. Due to the behavior of BWA with ambiguous reference states (*i.e.*, replacing N with one of G, A, T or C randomly), sites were masked with a random non-reference GATC nucleotide. The final AR5-like and E8-like homeolog references were restricted to genes with ≥150-bp of unmasked sequence (*n* = 6,698).

Allopolyploid transcriptome analysis

Paired-end, 100-bp reads from the Lp1 allopolyploid were trimmed dynamically as described above. Paired-end reads ≥50-bp from each of the two biological replicates were mapped independently to the AR5-like and E8-like homeolog references using BWA. Only uniquely mapping reads with no mismatches were accepted, and the numbers of reads mapping to the AR5-like and E8-like homeologs were counted for each gene. Mappings in which both read pairs aligned were counted as a single ‘match’.

Visualization

All datasets and read mappings were extensively checked for errors manually using Integrative Genomics Viewer (IGV) v. 2.0 [79]. For visual convenience, masked sites in the gene references were replaced with ‘N’, and visualization was performed against a gene reference containing only the ancestral SNPs.

**Supporting References**

83. Camacho C, Coulouris G, Avagyan V, Ma N, Papadopoulos J, et al. (2009) BLAST+: Architecture and applications. BMC Bioinformatics 10: 421.

84. Li H, Durbin R (2010) Fast and accurate long-read alignment with Burrows-Wheeler transform. Bioinformatics 26: 589-595.

85. Li H, Handsaker B, Wysoker A, Fennell T, Ruan J, et al. (2009) The sequence alignment/map format and SAMtools. Bioinformatics 25: 2078-2079.
