# Supplementary material for: An Interspecific Fungal Hybrid Reveals Cross-Kingdom Rules for Allopolyploid Gene Expression Patterns
Source: PLoS Genet. 2014 Mar 6;10(3):e1004180. doi: 10.1371/journal.pgen.1004180 (PMC3945203; doi:10.1371/journal.pgen.1004180)
Supplement: Table S1 — Lp1 contains E8 but not AR5 rDNA sequences. Table showing counts of reads from the parents and Lp1 that were mapped to parental ITS reference sequences. (DOCX) [file pgen.1004180.s009.docx]

**Table S1. Lp1 contains E8 but not AR5 rDNA sequence reads**

| **Transcriptome** | **ITS sequence mapped to** | **Replicate 1** | **Replicate 2** | **Total** |
| --- | --- | --- | --- | --- |
| E8 RNA-seq | E8 ITS | 1921 | 1325 | 3246 |
| AR5 RNA-seq | E8 ITS | 44 | 2 | 46 |
| **Lp1 RNA-seq** | **E8 ITS** | **4446** | **5026** | **9472** |
| E8 RNA-seq | AR5 ITS | 3 | 0 | 3 |
| AR5 RNA-seq | AR5 ITS | 2022 | 1870 | 3892 |
| **Lp1 RNA-seq** | **AR5 ITS** | **1** | **3** | **4** |

The number of reads mapping to the E8 ITS or the AR5 ITS are shown for the two replicates of the E8, AR5 and Lp1 transcriptome datasets. The Lp1 replicates each contain both paired end reads. Almost all Lp1 reads (bold) map to the E8 ITS. The number of Lp1 reads mapping to AR5 is less than the level seen in inter-parental cross mapping, suggesting that Lp1 is devoid of AR5-derived rDNA.
