# Supplementary material for: An Interspecific Fungal Hybrid Reveals Cross-Kingdom Rules for Allopolyploid Gene Expression Patterns
Source: PLoS Genet. 2014 Mar 6;10(3):e1004180. doi: 10.1371/journal.pgen.1004180 (PMC3945203; doi:10.1371/journal.pgen.1004180)
Supplement: Table S2 — PCR primers used in this study. (DOCX) [file pgen.1004180.s010.docx]

**Table S2. PCR primers used in this study.**

| **Primer** | **Sequence (5ʹ-3ʹ)** |
| --- | --- |
| 49910-left | TCCTCAACCAGAAGCAACAA |
| 49910-right | CTACGTTGCCGTCCATGTC |
| 50100-left | GACGGTGTTACCACGACACA |
| 50100-right | GTGATCCCTTTGTTGCGTCT |
| 83735-left | ACGTGTCATACTGGCGTGAT |
| 83735-right | CGATCCTAGAAGGCGAGAAG |
| 104800-left | TACGCCATCACATCCGTCAT |
| 104800-right | TGCATACAAGACGCCATACA |
| 52100-left | ATCCCACATCACTGCCTTTG |
| 52100-right | TGCTCTGCTCGATCATGGTT |
| 58000-left | CACAAGACATCGCCTCAAGA |
| 58000-right | GGCGTGAACTGGGACCTAC |
| 17210-left | GAACAGAGCGGGATGAGCCAAT |
| 17210-right | ATCTCCGCCGCATTCTTTGGCA |
