# Supplementary material for: An Interspecific Fungal Hybrid Reveals Cross-Kingdom Rules for Allopolyploid Gene Expression Patterns
Source: PLoS Genet. 2014 Mar 6;10(3):e1004180. doi: 10.1371/journal.pgen.1004180 (PMC3945203; doi:10.1371/journal.pgen.1004180)
Supplement: Text S1 — Dating the allopolyploidization event. Description of how the age range of Lp1 was estimated. (DOCX) [file pgen.1004180.s011.docx]

**Text S1. Dating the allopolyploidization event**

Lp1 is a naturally occurring allopolyploid that was first isolated from the field in the early 1990s. However, the timing of the allopolyploidization event is not known. To provide some estimate of this time, we looked at the number of SNPs on the shortest parental branch, in this case the AR5 lineage, as this provides an upper bound on the allopolyploidization time (**Figure S6**). We identified 2,183 SNPs that occur in AR5, but not Lp1 (‘AR5-unique’). Given an estimated lower bound on the mutation rate of ~1x10^-9^/site/year (0.9-16.7x10^-9^/site/year for protein coding genes in Eurotiomycete fungi; [81]) and a cumulative haploid sequence length of 7,123,190 bp in the masked reference, AR5 would be expected to accumulate 2,183 new SNPs in no more than 306,464 years. However, it is possible that AR5 is not the exact parent of Lp1, so this age is only provided as an upper bound on the allopolyploidization time.

It is not possible to place a lower bound on the allopolyploidization event. However, there is evidence to suggest that Lp1 is not just a transient interspecific organism that formed immediately prior to isolation. First, we identified a number of gene deletion events that have occurred since the allopolyploidization event (**Figure 4**). Second, previous results suggested that both the mitochondrial DNA and the ribosomal DNA (rDNA) repeats in Lp1 derive exclusively from one parent: rDNA from the E8 progenitor and mtDNA from the AR5 parent. We identified diagnostic substitutions at three sites that distinguish AR5 and E8 mitochondrial DNA. At all three diagnostic positions, all Lp1 sequence reads carry the AR5-like variant DNA (**Figure S7**), thus confirming that mitochondrial homoplasmy has occurred in Lp1. However, given that mitochondrial homoplasmy can occur over short timeframes [86], this provides only weak evidence for Lp1 being a well-established allopolyploid. It was reported that the E8 rDNA has physically replaced AR5 rDNA across the genome through a process known as interchromosomal homogenization [57]. This is thought to be gradual process [62,63,87,88], thus suggesting the allopolyploidization event is not very recent. Consequently, we used our transcriptome data to determine how efficient removal of AR5 rDNA sequences has been. Although total RNA was subjected to polyA selection, there is usually a significant amount of contamination from the highly abundant rRNA in these libraries. We mapped the transcriptome data to both E8 and AR5 ITS sequences, as the ITS shows sufficient variation to distinguish the two parental types. Almost all Lp1 reads map to E8, not AR5 (**Table S1**). Some Lp1 reads map to AR5, but fewer than the control mapping where one parental transcriptome was mapped to the other parent’s ITS. This suggests that a low level of cross mapping occurs due to sequence errors that spuriously match the other parent’s ITS region. Therefore, we conclude that probably no AR5-like rDNA copies remain in the Lp1 genome. Together with the stable maintenance of Lp1 in culture since its isolation in the 1990s and the widespread identification of epichloë allopolyploids in the field [5] despite the difficulty in creating these artificially in the lab [89,90], these results suggest that Lp1 is not just a transient organism, but is instead an established allopolyploid that arose sometime within the past three hundred thousand years.

**Supporting References**

86. MacAlpine DM, Kolesar J, Okamoto K, Butow RA, Perlman PS (2001) Replication and preferential inheritance of hypersuppressive petite mitochondrial DNA. EMBO J 20: 1807-1817.

87. Coen E, Strachan T, Dover G (1982) Dynamics of concerted evolution of ribosomal DNA and histone gene families in the *melanogaster* species subgroup of *Drosophila*. J Mol Biol 158: 17-35.

88. Kovarik A, Pires JC, Leitch AR, Lim KY, Sherwood AM, et al. (2005) Rapid concerted evolution of nuclear ribosomal DNA in two *Tragopogon* allopolyploids of recent and recurrent origin. Genetics 169: 931-944.

89. Christensen MJ, Simpson WR, Al Samarrai T (2000) Infection of tall fescue and perennial ryegrass plants by combinations of different *Neotyphodium* endophytes. Mycological Res 104: 974-978.

90. Chung KR, Schardl CL (1997) Vegetative compatibility between and within *Epichloë* species. Mycologia 89: 558-565.
