# Supplementary material for: An Interspecific Fungal Hybrid Reveals Cross-Kingdom Rules for Allopolyploid Gene Expression Patterns
Source: PLoS Genet. 2014 Mar 6;10(3):e1004180. doi: 10.1371/journal.pgen.1004180 (PMC3945203; doi:10.1371/journal.pgen.1004180)
Supplement: Text S2 — GO-slim analysis. Description of how the different gene sets were analyzed using GO-slim to look for evidence of selection having shaped the transcriptional response to allopolyploidy. (DOCX) [file pgen.1004180.s012.docx]

**Text S2. GO-Slim analysis**

To perform gene ontology (GO) analyses [91], GO annotation files of all genes and the various subsets of genes were analyzed using the GO-slim online tool of AgBase (http://www.agbase.msstate.edu/cgi-bin/tools/goslimviewer_select.pl), using the “Generic” GO-slim set [92]. GO-slim groups genes into a reduced set of gene ontology categories at higher hierarchical levels. The results were analyzed in Excel. Ratios of the number of genes in the ‘all’ gene set versus the gene set of interest were calculated, and those differing by at least 1.5-fold were plotted (alongside the raw number of genes). The gene sets analyzed are: genes with no change in expression; homeolog expression blending genes; homeolog expression bias genes; homeolog expression reversal genes; and all differentially expressed Lp1genes (**Figure S3**).

To look for evidence of selection on the changes in gene expression, the five different sets of genes for which GO-slim analyses were performed were divided into two classes. If gene expression is responding to selective forces, then genes showing patterns of gene expression change are predicted to be enriched for GO-slim categories that relate to adaption to changing environments, while those that are recalcitrant to gene expression change are predicted to be enriched for housekeeping categories. Therefore the gene sets were divided into these two classes as follows:

Class I includes gene sets for which expression is not equal, and consists of: genes where differential expression has occurred in the allopolyploid (homeolog expression bias); genes where the direction of differential expression has been reversed in the allopolyploid (homeolog expression reversal); and all genes that are differentially expressed in the allopolyploid (Lp1 differentially expressed).

Class II includes gene sets where expression patterns are conservative in Lp1 and consists of: genes that show no expression differences between the allopolyploid and parents (no change); and genes that have lost differential expression in the allopolyploid (homeolog expression blending).

We reasoned that gene sets within a class should show reciprocal enrichment/depletion patterns for GO-slim categories with those gene sets from the other class. Therefore we looked for GO-slim categories that have an altered ratio for at least two of the five different gene sets. In the 33 such GO-slim categories identified, we then looked to see which of these had a consistent pattern with respect to the two classes (e.g. a category is over-represented in class I gene sets, but under-represented in class II gene sets). Approximately half the categories (n = 16) showed such a consistent pattern between the two classes. We further reasoned that if the signature of over/under-representation comes from selective forces acting on gene expression, the categories in the first class should be involved in adaptation, while those in the second class should be housekeeping. Therefore we divided these 16 categories into those that show over-representation in class I sets and/or under-representation in class II sets; or the reverse pattern. There are 5 categories in the first division: “cytoskeleton-dependent intracellular transport”, “extracellular matrix organization”, “lysosome”, “secondary metabolic process”, and “sulfur compound metabolic process”. Some of these are clearly categories involved in adaptation to the environment, most notably “secondary metabolic processes”, as the diversity in type and amount of secondary metabolites in endophytes has been well-documented [59], although a number of these are not expressed in culture, but also “lysosome” and possibly “sulfur compound metabolic process” and “extracellular matrix organization”. However, “cytoskeleton-dependent intracellular transport” is expected to include housekeeping genes. There are 11 categories in the second division: “cell junction organization”, “circulatory system process”, “external encapsulating structure”, “histone binding”, “nucleolus”, “pigmentation”, “ribonucleoprotein complex assembly”, “ribosome”, “rRNA binding”, “structural molecule activity”, and “symbiosis, encompassing mutualism through parasitism”. Many of these GO-slim categories are clearly housekeeping in nature, as expected. However, some are not obviously housekeeping groups (e.g. “pigmentation”) or are not expected in these fungi (e.g. “circulatory system process”). Further, “symbiosis, encompassing mutualism through parasitism” might also be considered adaptive. It should also be noted over half (9/16) of these categories represent fewer than 50 genes in the total gene set. Deviation from expected numbers can occur due to small changes in gene number in categories with few genes, thus making these categories with small gene numbers more prone to spurious effects.

In summary, the GO-slim analyses show that some changes in gene expression are consistent with selection for either conservative or non-conservative gene expression profiles, but that there are a number where the patterns we observe are the opposite to those predicted if the responses are being shaped by selection. Furthermore, in many cases the sets that show expression changes consistent with selection consist of a small number of genes. Therefore, we conclude that there is little evidence to suggest that selective forces have shaped the transcriptional response for the majority of genes in our analysis.

**Supporting References**

91. Gene Ontology Consortium (2012) The Gene Ontology: Enhancements for 2011. Nucleic Acids Res 40: D559-564.

92. McCarthy FM, Wang N, Magee GB, Nanduri B, Lawrence ML, et al. (2006) AgBase: a functional genomics resource for agriculture. BMC Genomics 7: 229.
