# Supplementary material for: An Interspecific Fungal Hybrid Reveals Cross-Kingdom Rules for Allopolyploid Gene Expression Patterns
Source: PLoS Genet. 2014 Mar 6;10(3):e1004180. doi: 10.1371/journal.pgen.1004180 (PMC3945203; doi:10.1371/journal.pgen.1004180)
Supplement: Text S3 — Determining the proportion of genes that have evolved varying degrees of transcriptional independence since speciation of the parents. Description of the logic and method used to calculate the percent range of genes that have undergone full, partial and no transcriptional independence since the speciation of the two parent species prior to the allopolyploidization event. (DOCX) [file pgen.1004180.s013.docx]

**Text S3. Determining the proportion of genes that have evolved varying degrees of transcriptional independence since speciation of the parents**

We propose that the bulk of the transcriptional responses in the allopolyploid represent changes in the regulatory modulons of genes that have occurred in the parental species in the time since they speciated. These regulatory changes mean that different orthologs have evolved different levels of transcriptional independence, and these are revealed by the transcriptional patterns seen in Lp1. To provide estimates of the proportions of genes where the orthologs have evolved transcriptional independence, partial independence and have not evolved independence, we have used the number of genes that show simple inheritance of biased expression, homeolog expression bias, and homeolog expression blending, respectively (see **Figure 8**).

However, a limitation of this analysis is that a large number of genes show no expression difference in either Lp1 or the parents, and whether or not these have evolved any transcriptional independence cannot be determined. To help provide an estimate of genes with varying levels of transcriptional independence that includes this large class of genes, we used information on the proportions of genes from above. To estimate a lower bound for the proportion of genes having evolved transcriptional independence, we assumed that none of these non-biased genes have evolved independence. To estimate the upper bound, we assumed that these non-biased genes have evolved the same proportions of independence/no independence as found for the other, biased genes. Therefore the proportions of genes in these three transcriptional independence categories are reported as a range in the main text that reflects these lower and upper bounds.
